# Extracellular RNA in viral–host interactions: Thinking outside the cell

**DOI:** 10.1002/wrna.1535

**Published:** 2019-04-08

**Authors:** Sarah Ressel, Adelina Rosca, Katrina Gordon, Amy H. Buck

**Affiliations:** ^1^ Institute of Immunology and Infection Research, School of Biological Sciences University of Edinburgh Edinburgh UK; ^2^ Department of Virology Carol Davila University of Medicine and Pharmacy Bucharest Romania

**Keywords:** extracellular RNA, extracellular vesicle, host–pathogen, microRNA, RNA interference

## Abstract

Small RNAs and their associated RNA interference (RNAi) pathways underpin diverse mechanisms of gene regulation and genome defense across all three kingdoms of life and are integral to virus–host interactions. In plants, fungi and many animals, an ancestral RNAi pathway exists as a host defense mechanism whereby viral double‐stranded RNA is processed to small RNAs that enable recognition and degradation of the virus. While this antiviral RNAi pathway is not generally thought to be present in mammals, other RNAi mechanisms can influence infection through both viral‐ and host‐derived small RNAs. Furthermore, a burgeoning body of data suggests that small RNAs in mammals can function in a non‐cell autonomous manner to play various roles in cell‐to‐cell communication and disease through their transport in extracellular vesicles. While vesicular small RNAs have not been proposed as an antiviral defense pathway per se, there is increasing evidence that the export of host‐ or viral‐derived RNAs from infected cells can influence various aspects of the infection process. This review discusses the current knowledge of extracellular RNA functions in viral infection and the technical challenges surrounding this field of research.

This article is categorized under:

Regulatory RNAs/RNAi/Riboswitches > Regulatory RNAs
RNA in Disease and Development > RNA in Disease
Regulatory RNAs/RNAi/Riboswitches > RNAi: Mechanisms of Action

## 1 SMALL RNA‐MEDIATED GENE REGULATION IN EUKARYOTES

Small RNA‐mediated gene regulation is a central component of how eukaryotic organisms develop, adapt, and protect themselves from viruses and transposable elements. Small RNAs usually operate by guiding an Argonaute (AGO) protein to another nucleic acid target through Watson–Crick base pair complementarity (Azlan, Dzaki, & Azzam, 2016). These interactions lead to gene silencing, which can involve transcriptional, posttranscriptional or epigenetic mechanisms, depending on the properties of the small RNA, AGO protein and target in a given organism (Munshi, Mohan, & Ahuja, 2016). In animals, there are two prominent conserved RNA interference (RNAi) mechanisms: (a) the posttranscriptional regulation of gene expression by microRNAs (miRNAs) and (b) the posttranscriptional and transcriptional silencing of transposable elements by the piwi‐interacting RNA (piRNA) pathway (Bartel, 2018; Baulcombe, 2004; Hannon, 2002). Another important RNAi pathway that is thought to be ancestral is “antiviral RNAi,” where viral dsRNA is processed into small interfering RNAs (siRNAs) that bind the complementary sequences to degrade viral genomes (Ding & Voinnet, 2007). Antiviral RNAi is found in plants, fungi and many animals, and can operate through non‐cell autonomous mechanisms, spreading the signal from infected cells to distant sites in the organism (Obbard, Gordon, Buck, & Jiggins, 2009). The concept of mobile small RNA, therefore, goes hand‐in‐hand with antiviral RNAi in many organisms and in plants these discoveries date back two decades (Hamilton & Baulcombe, 1999). Evidence suggests antiviral RNAi exists in pluripotent cells in mammals (Ding, Han, Wang, & Li, 2018) but type I interferons (IFNs) are thought to be the main mechanism of antiviral defense in differentiated cells (Pare & Sullivan, 2014; tenOever, 2017).

Beyond the debated existence of antiviral RNAi in mammals, other RNAi pathways can influence the infection process and these can involve both viral‐ or host‐derived small RNA guides. Here we detail the functions of these diverse small RNAs inside viral‐infected cells. We then explore the recent literature suggesting that the transmission of small RNAs between cells plays important functional roles in viral infections. We review the mechanisms for RNA transport between cells and highlight the technical challenges for characterizing these pathways as well as new methodologies required to advance the field.

## 2 VIRAL‐ AND HOST‐DERIVED miRNAs INSIDE THE CELL: A DIVERSE RANGE OF FUNCTIONS

miRNAs are the most extensively studied class of small RNA associated with viral–host interactions in mammals. The field of miRNA research in animals evolved from characterization of their important roles in development and revolutionized our understanding of posttranscriptional gene regulation (Alvarez‐Garcia & Miska, 2005). Over the last decade it has become clear that miRNAs are involved in regulating virtually every process in the cell and are important players in cancers and other diseases (Mendell & Olson, 2012). miRNAs are a highly conserved class of endogenous small RNA that derive from hairpin transcripts, generally produced by RNA polymerase II. The canonical biogenesis pathway involves processing of the primary miRNA transcripts by the RNase III enzyme Drosha and cofactors (also called the microprocessor complex) in the nucleus. The resulting precursor miRNA is then exported to the cytoplasm and further processed by another RNase III enzyme, Dicer, resulting in a ~22 nucleotide (nt) double‐stranded miRNA duplex which is loaded into the RNA‐induced silencing complex (RISC). The guide strand of the miRNA associates with an AGO protein in the RISC while the passenger strand is degraded (Ha & Kim, 2014). The canonical mechanism of action of a miRNA is to bind to specific messenger RNAs (mRNAs) and inhibit translation and/or accelerate de‐adenylation (Bartel, 2018). Target recognition largely occurs via complementary base pairing of the miRNA with the mRNA target (Lewis, Burge, & Bartel, 2005). Most evolutionarily conserved target sites are found in the 3′ untranslated (3′UTR) of mRNAs and involve pairing with nucleotides 2–8 from the 5′ end of the miRNA, termed the “seed” (Brennecke, Stark, Russell, & Cohen, 2005; Lewis, Shih, Jones‐Rhoades, Bartel, & Burge, 2003). In 2004, it was shown that certain herpesviruses can hijack the host miRNA biogenesis machinery to express their own miRNAs (Pfeffer et al., 2004). Subsequently, viral miRNAs have been identified in several other viral families, including Adenoviridae, Flavirividae, Orthomyxoviridae, and Retroviridae (Fruci, Rota, & Gallo, 2017; Kincaid & Sullivan, 2012; Tycowski et al., 2015). These studies have also revealed novel, noncanonical pathways for viral miRNA biogenesis which are independent of Drosha (Xie & Steitz, 2014).

### 2.1 Functional roles of viral miRNAs inside cells

**Figure 1:**
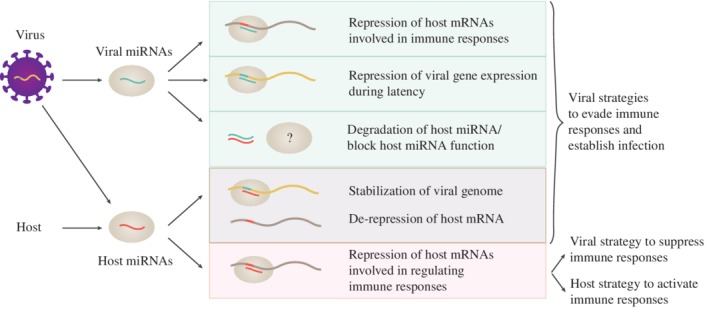
Functions of microRNA (miRNA)–target interactions inside an infected cell. Viruses encode miRNAs which can regulate host messenger RNAs (mRNAs), viral mRNAs, and host miRNAs to promote infection. At the same time infections lead to changes in host miRNA expression which influences immune responses

Viral‐encoded miRNAs can have diverse functions during infection through their regulation of viral or host genes, depicted in Figure 1 (Kincaid & Sullivan, 2012; Tycowski et al., 2015). For example, an early report demonstrated that polyomaviruses produce miRNAs which repress viral T antigen at late stages of infection as a mechanism of immune evasion (Sullivan, Grundhoff, Tevethia, Pipas, & Ganem, 2005). In all of the diverse herpesvirus subfamilies, viral miRNAs have been shown to target the viral immediate‐early trans‐activator proteins that drive viral replication. It is proposed that this regulation might be key to maintaining latency while setting the appropriate threshold to respond to reactivation signals (Grey, 2015). At the same time, viral miRNAs can also directly interact with host mRNAs to regulate cellular networks that promote persistence, replication, and/or immune evasion (Bruscella et al., 2017; Cullen, 2006). For example, the Epstein–Barr virus (EBV)‐encoded miRNA BART6 inhibits the induction of antiviral immune responses by targeting genes of the retinoic acid‐inducible gene I (RIG‐I) signaling pathway (Lu et al., 2017), while miRNAs derived from the oncogenic human Kaposi's sarcoma‐associated herpesvirus (KSHV) enhance the survival of infected cells by inhibiting genes involved in cell cycle and/or apoptosis (Gottwein & Cullen, 2010; Liu, Happel, & Ziegelbauer, 2017).

### 2.2 Enhancement of infection by direct interactions between cellular host miRNAs and viruses

Cellular host miRNAs can also impact viral infection through a range of diverse targets and in some cases this involves direct interaction with viral genomes (Figure 1). For example, an early report showed that hepatitis C virus (HCV) has evolved a binding site in its genome for miR‐122, a miRNA naturally highly expressed in the liver (Jopling, Yi, Lancaster, Lemon, & Sarnow, 2005). This interaction with miR‐122 stabilizes the HCV genome and also leads to a derepression of miR‐122 host targets, which are associated with lipid metabolism (Sarnow & Sagan, 2016). Many other host miRNA binding sites have since been reported in the genomes of diverse viral families (Trobaugh & Klimstra, 2017). In contrast to the miR‐122‐HCV example, interaction with host miRNAs can also lead to a suppression of viral translation and replication. For example, the myeloid expressed miRNA, miR‐142‐3p, binds to the 3′ untranslated region of eastern equine encephalitis virus, suppressing its replication in myeloid cells. This interaction appears to be advantageous to the virus, leading to reduced induction of the host innate immune response (Trobaugh et al., 2014). Indeed, it is expected that rapidly evolving viruses would not maintain genomic binding sites for host miRNAs if the interactions were not beneficial for the virus. Host miRNA–virus interactions, therefore, are suggested as another viral mechanism to escape detection and clearance by the immune system and enable persistent infections (Mahajan, Drake, & Chen, 2009).

Some viruses have also evolved to interact with specific host miRNAs in a way that leads to degradation of the miRNA (McCaskill, Praihirunkit, Sharp, & Buck, 2015). For example, two different herpesviruses, murine cytomegalovirus and herpesvirus saimiri (HVS), have evolved distinct noncoding RNAs that interact with miR‐27 and mediate its degradation (Cazalla, Yario, Steitz, & Steitz, 2010; Libri et al., 2012; Marcinowski et al., 2012; Guo, Oei, & Steitz, 2015). It is assumed that the function of this interaction is to block the natural regulatory mechanism of miR‐27 (e.g., leading to an increase in the host targets of miR‐27). Indeed, it was further shown by the Steitz lab that miR‐27 is a repressor of T cell activation and proposed that the degradation of miR‐27 by HVS enables constitutive activation of HVS‐infected T cells, a key cell type for latency (Guo, Riley, Iwasaki, & Steitz, 2014). Many additional host miRNA–virus interactions that lead to deregulation of the host miRNAs and/or inhibit their functional capacity have since been reported, underscoring the idea that an individual miRNA can be important enough for a virus to devote genome space to its inhibition.

### 2.3 Cellular miRNA regulation and functions in defense

Cellular (host) miRNAs regulate nearly every signaling pathway inside the cells and they work in concert with transcription factors to control initiation, maintenance, and resolution of cell response to injury and stress (Mendell & Olson, 2012). The functions of host miRNAs inside infected cells have been reviewed in depth recently (Girardi, López, & Pfeffer, 2018; Trobaugh & Klimstra, 2017), which we will not duplicate. We note, however, that it is not always trivial to understand whether the up‐ or down‐regulation of a given miRNA upon infection is driven by a viral or host mechanism. For example, multiple viral infections, including enterovirus 71 (EV71) and dengue virus, lead to an up‐regulation of miRNA‐146 (Ho et al., 2014; S. Wu et al., 2013). This miRNA is part of a negative feedback loop to suppress the IFN response and miR‐146 targets the tumor necrosis factor (TNF) receptor associated factor 6 (Ho et al., 2014; S. Wu et al., 2013; Yoshida et al., 2008). It is therefore possible that the upregulation of miR‐146 upon infection is a natural host mechanism to control the response, but this may also be modulated/enhanced by the virus. In order to interpret the pro‐ or anti‐viral functions of miRNAs that are dysregulated upon infection it is highly relevant to understand whether and how the infection also leads to changes in the export of miRNAs, and their transmission to uninfected cells (explored further below).

At the same time, it is worth noting that some viral infections can lead to suppression of the entire miRNA pathway inside of cells. As a viral mechanism, this would mean sacrificing the ability to exploit the miRNA pathway with viral miRNAs in the infected cell, with the benefit that the antiviral properties of some host miRNAs would be inhibited. For example, the poly(A) polymerase of mammalian poxviruses polyadenylates host miRNAs resulting in their degradation (Backes et al., 2012), although this does not affect all miRNAs equally (Buck et al., 2015). Suppression of the host miRNA pathway upon infection could also represent a host mechanism for boosting the IFN response, since certain miRNAs function as negative regulators of the IFN pathway. In particular, the recognition of viral RNA results in the inhibition of miRNA biogenesis by suppressing the activity of the microprocessor complex. This downregulation leads to the derepression of *IFNB1* mRNA and several IFN‐stimulated genes (ISGs) (Witteveldt, Ivens, & Macias, 2018) which have been reported to be repressed by miRNAs under normal conditions (Hsu et al., 2017; Witwer, Sisk, Gama, & Clements, 2010). Similarly, ISGs can be activated by reducing the silencing activity of RISC through poly‐ADP‐ribosylation which also leads to derepression of miRNA targets (Seo et al., 2013).

From the above examples it is clear that there are a multitude of possibilities for how cellular miRNA levels impact an infected cell, and how individual miRNAs or the miRNA machinery can be modulated during infection (not all of which are covered here). At the same time, emerging data suggest viral infections also impact the secretion of miRNAs from infected cells, and this can include both viral‐ and host‐derived miRNAs. In this review, we consider the extracellular realm when thinking about the role of miRNAs and other small RNAs in viral infection.

## 3 EXTRACELLULAR SMALL RNAs IN MAMMALS

It has long been known that various RNA species exist outside of cells in different body fluids, and can be released from cells and internalized by other cells under physiological conditions (de Candia, De Rosa, Casiraghi, & Matarese, 2016; Hoy & Buck, 2012; Valadi et al., 2007). Studies from the 1960s and 1970s showed that extracellular RNA is transferred between fibroblast cells (Kolodny, 1971, 1972) and from macrophages to lymphocytes (Fishman, Hammerstrom, & Bond, 1963). The transfer of RNA was also linked to induction of T cell‐specific antigen in bone marrow lymphocytes (Archer, 1978). However, the mechanisms and identity of RNA species were not examined. Another report suggested highly methylated RNA is released from a diverse range of cells under physiological conditions, in a manner that is not associated with cell death (Stroun et al., 1978). Despite these intriguing early findings, little was reported on extracellular RNA until 2008, when seminal papers showed that miRNAs are present in body fluids outside of cells (X. Chen et al., 2008; Chim et al., 2008; Lawrie et al., 2008; Mitchell et al., 2008). Furthermore, since the profile of miRNAs in biofluids was shown to change in pathological conditions, this stimulated extensive commercial interest in their biomarker capacity (Fritz et al., 2016; Sohel, 2016). Around the same time, in vitro studies showed that miRNAs and mRNAs are exported from cells in extracellular vesicles (EVs). These can transfer the RNAs to other cells where they mediate changes in gene expression (Skog et al., 2008; Valadi et al., 2007). The combination of these discoveries has led to an explosion of interest in the translational applications of EVs, and their miRNA cargos, over the last 10 years. However, relatively little is known about the processes of RNA export and import and how this is controlled in the cell. The majority of research in this area has focused on understanding EV composition and function.

## 4 EXTRACELLULAR VESICLES—FORM AND FUNCTION

The term EVs refers to small organelles enclosed by a lipid bilayer membrane that are found outside cells. These vesicles can be broadly divided into three subclasses based on their origins inside cells. Apoptotic bodies are released from cells undergoing apoptosis. Microvesicles (also microparticles or ectosomes) are 100–1,000 nm vesicles released by budding from the plasma membrane, while exosomes are small vesicles (~100 nm) of endosomal origin which are released by fusion of multivesicular endosomes (MVEs) with the plasma membrane (Colombo, Raposo, & Théry, 2014; Yáñez‐Mó et al., 2015). However, many studies have used the term exosomes without showing evidence for subcellular origin. Moreover, it has become clear that widely used isolation methods (such as differential centrifugation) result in mixed EV populations (Konoshenko, Lekchnov, Vlassov, & Laktionov, 2018; Lötvall et al., 2014) and EVs that are small (<100 nm) are not exclusively exosomes (Kowal et al., 2016). For this reason, we will only use the term EVs in this review, even if the original paper refers to these as exosomes. EVs have gained popularity in the last decade in part due to the fact that they can shuttle RNAs between cells and also based on extensive literature showing their roles in many pathological contexts, including cancer (Becker et al., 2016; De Toro, Herschlik, Waldner, & Mongini, 2015).

### 4.1 Extracellular vesicles and the Trojan Horse hypothesis

There are many types of viral cargo that can be packaged in EVs for transport to other cells, including viruses themselves. Indeed, it has long been hypothesized that viruses hijack cellular pathways of intercellular trafficking not only for biogenesis of new viral particles but also as an important mode of infection. This “Trojan exosome hypothesis” proposed that viruses can take advantage of cell‐encoded vesicle trafficking systems to move between cells without the need of viral receptors for cell entry and in order to evade detection by the immune system (Gould, Booth, & Hildreth, 2003). Many reports have demonstrated that viruses can package full length viral genomes or mRNA in EVs, which are able to replicate and and/or contribute to the establishment of infections in recipient cells (Bukong, Momen‐Heravi, Kodys, Bala, & Szabo, 2014; Chivero et al., 2014; Columba Cabezas & Federico, 2013; Cosset & Dreux, 2014; Dreux et al., 2012; Fu et al., 2017; Jaworski et al., 2014; Mao et al., 2016; Mori et al., 2008; Ramakrishnaiah et al., 2013). The use of EVs for transmission of viral genomes or mRNAs is reviewed further in van Dongen, Masoumi, Witwer, and Pegtel (2016) and may be particularly important for naked virus transmission to occur without cell lysis (van der Grein, Defourny, Slot, & Nolte‐'t Hoen, 2018). Here we focus on describing the small RNAs that are exported from infected cells and the current thinking on their functions. We cover the properties and functions of extracellular small RNAs that have been reported in a viral context, starting with a focus on miRNAs and how their transfer in EVs to recipient cells might impact infection through canonical (gene silencing) or noncanonical (Toll‐like receptor [TLR] activation) mechanisms.

## 5 DUAL ROLE OF miRNAs IN EVs: ACTIVATORS AND REPRESSORS OF IMMUNE RESPONSES

**Figure 2:**
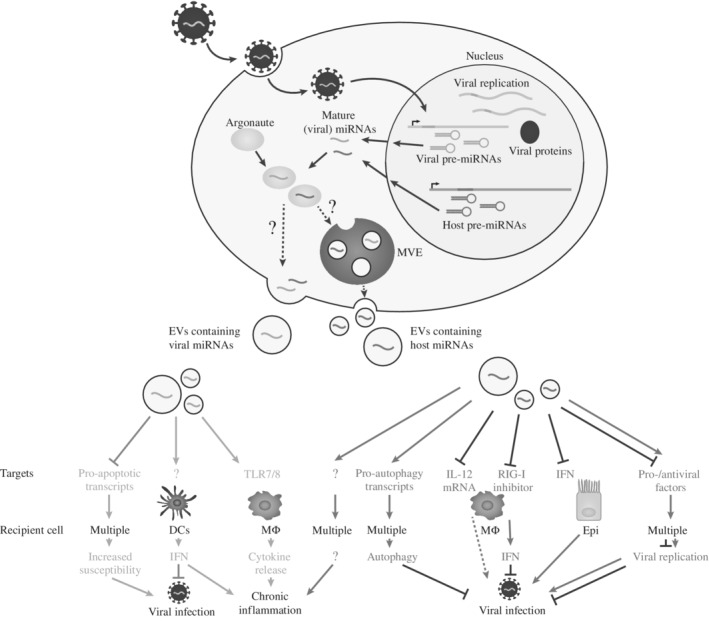
Functional modes of secreted (viral) microRNAs (miRNAs). Host‐ and viral‐encoded miRNAs which are transcribed in the nucleus and processed in the nucleus and cytoplasm can be exported from the cell in extracellular vesicles (EVs). Export pathways involve multivesicular endosomes (MVEs) or budding from the plasma membrane. Viral miRNAs when transferred to recipient cells can promote/inhibit infection by regulating gene transcripts. Examples are shown involving viral miRNA transfer to dendritic cells (DCs) or macrophages (MΦ) that can lead to chronic inflammation through the activation of toll‐like receptors (TLRs). Host miRNAs transmitted from infected to uninfected cells can either enhance or suppress viral infections and/or contribute to chronic inflammation. Antiviral immune responses can be suppressed by host miRNAs that target the interferon (IFN) pathway in epithelial cells (Epi) or through inhibition of macrophage activation. On the other hand, host miRNAs can activate the IFN response by suppressing negative regulators of the retinoic acid‐inducible gene I (RIG‐I). Other mechanisms of host defense include the activation of autophagy

In the last 5 years several reports have demonstrated that both host and viral miRNAs are transferred from infected cells to other (noninfected) cells and this miRNA trafficking has been proposed to facilitate infection through the regulation of immune responses (Kouwaki, Okamoto, Tsukamoto, Fukushima, & Oshiumi, 2017; Laganà et al., 2013). A detailed list of findings from various studies is provided in Table 1 with several examples depicted in Figure 2. In the following section we detail research on viral small RNAs released from cells infected with EBV and human immunodeficiency virus (HIV), where there is the largest body of literature to date.

**Table 1:** Virus‐ or host‐derived extracellular small RNAs and their proposed functions during infection

Viral infection	Viral small RNA	Target/function	Viral replication	References
EBV	BART, BHRF1‐3	BHRF1‐3: Downregulation of *cxcl11* gene in DCs BART: Interaction with LMP1 (viral protein)	↑?	Pegtel et al. (2010)
	5′pppEBER1	Activation of DCs	?	Baglio et al. (2016)
HIV‐1	TAR miRNAs	Induction of NF‐κB pathway, facilitate viral infection of uninfected cells	↑	Narayanan et al. (2013); Sampey et al. (2016)
	vmiR‐88, vmiR99	Release of TNFα through activation of TLR8 signaling	?	Bernard et al. (2014)
KSHV	miR‐K12‐10a‐3p, K12‐4‐3p, K12‐8‐3p	Induction of aerobic glycolysis, reduced mitochondria biogenesis	↑?	Yogev et al. (2017)

Viral infection	Host miRNA	Target/function	Viral replication	References
HCV	miR‐19a	Inhibition of SOC3, role in fibrosis	?	Devhare et al. (2017)
	miR‐29	↑?	↓	Zhou et al. (2016)
	miR‐122	Viral genome stabilization	↑	Ramakrishnaiah et al. (2013); Bukong et al. (2014)
HBV	miR‐29, miR‐21	Inhibition of IL‐12 in macrophages	↑	Kouwaki et al. (2016)
	miR‐122	Viral genome stabilization? Role in replication?	↑	Novellino et al. (2012); W. Wang, Li, Zhang, et al., 2016
IAV	miR‐483	Activation of RIG‐I pathway	↓	Maemura et al. (2018)
	miR‐17	Inhibition of antiviral factor Mx1	↑	Scheller et al. (2018)
PV, VV, HSV‐1	C19MC cluster	Induction of autophagy	↓	Delorme‐Axford et al. (2013)
hCMV	C19MC cluster	?	↑	Delorme‐Axford et al. (2013)
EV71	miR‐146a	Inhibition of IFN immune response	↑	Fu et al. (2017)
REV + ALV‐J	miR‐184, miR‐146, miR‐155 (all ↑), miR‐3538 (↓)	?	↑?	Zhou et al. (2018)
JEV	let‐7a/b	Neuronal damage and release of proinflammatory cytokines	?	Mukherjee et al. (2018)
HIV‐1	Multiple miRNAs	Inhibition of immune response due to export of miRNA	↑	Aqil et al. (2014)

*Note*. ↓, inhibited; ↑, enhanced; ?, unknown.

Abbreviation: 5′pppEBER1, 5′ polyphosphorylated EBV‐encoded small RNA; ALV‐J, avian leukosis virus subgroup J; C19MC, chromosome 19 miRNA cluster; DC, dendritic cells; EBV, Epstein–Barr virus; EV71, enterovirus 71; HBV, hepatitis B virus; hCMV, XXX; HCV, Hepatitis C virus; HIV‐1, human immunodeficiency virus 1; HSV‐1, herpes simplex virus 1; IAV, Influenza A virus; IFN, interferon; JEV, Japanese Encephalitis virus; KSHV, Kaposi's sarcoma‐associated herpesvirus; PV, poliovirus; REV, reticuloendotheliosis virus; RIG‐I, retinoic acid‐inducible gene I; TNFα, tumor necrosis factor α; VV, vaccinia virus.

### 5.1 Viral‐derived small RNAs in EVs: Gene silencers and TLR activators

#### 5.1.1 Epstein–Barr virus

An early report from 2010 showed that EVs released from EBV‐infected B cells contain viral miRNAs that are internalized by recipient monocyte‐derived dendritic cells (DCs). The authors showed proof‐of‐concept with reporter assays that EVs transfer viral miRNAs from infected cells to other cells and demonstrated the immune‐stimulatory gene *CXCL11*, a target of the viral miRNA, was suppressed in recipient cells (Pegtel et al., 2010). A subsequent study by the same group showed that other classes of viral small RNAs from EBV‐infected cells are also exported and can be transferred to recipient cells where they can activate immune responses. Specifically, 5′ polyphosphorylated EBV‐encoded small RNA (EBER1) is transferred via EVs to naive DCs which drive of antiviral immunity (Baglio et al., 2016). It is expected that the transfer of 5′ polyphosphorylated RNAs in EVs might be a broad mechanism for “warning” cells of stress and infection. For example, transfer of naked (not bound to protein) 7S RNA (which contains a 5′ triphosphate) in EVs from stromal cells activates RIG‐I in recipient cancer cells, linking inflammation and cancer progression (Nabet et al., 2017). In EBV infection, immune stimulation by EV‐imported RNA is thought to aid containment of the virus during the primary infection but can lead to chronic inflammation during latency (Baglio et al., 2016). These examples suggest multiple classes of viral‐derived small RNA can influence noninfected host cells, through canonical and noncanonical mechanisms. Further work is required to understand whether the release of particular viral RNAs is specific to certain cell types and stages of infection (lytic vs. latent) and the extent to which these can signal to near or distant cell types in vivo.

#### 5.1.2 Human immunodeficiency virus

Small RNAs derived from the HIV genome have been reported both in intra‐ and extra‐cellular contexts (Barclay et al., 2017; Bernard et al., 2014; Harwig, Jongejan, van Kampen, Berkhout, & Das, 2016; Klase et al., 2007, 2009; Narayanan et al., 2013; Narayanan, Kehn‐Hall, Bailey, & Kashanchi, 2011; Ouellet et al., 2008; Sampey et al., 2016; Schopman et al., 2012; Yeung et al., 2009). In particular, multiple studies showed that HIV‐1 trans‐activation response element RNA (TAR) can be processed into two miRNA‐like molecules, 5′ TAR and 3′ TAR (Klase et al., 2007, 2009; Narayanan et al., 2011; Ouellet et al., 2008). Both 5′ and 3′ TAR, together with the longer TAR RNA have been found in EVs isolated from culture supernatant of infected cells and patient sera (Narayanan et al., 2013). The transfer of TAR RNAs from infected to uninfected cells via EVs leads to the inhibition of pro‐apoptotic transcripts, thus preventing programmed cell death in the recipient cells and leaving them susceptible to HIV‐1 infection (Narayanan et al., 2013). Although the authors could not establish the exact mechanism of action or the actual involvement of the TAR RNAs, the fact that HIV‐derived RNAs have been detected in patient serum is intriguing, as it indicates release and potential functions in vivo.

Additionally, 5′ and 3′ TAR contained in EVs have been proposed to interact with TLRs 7 and 8 in recipient cells, to activate NF‐κB and regulate cytokine production during latency (Sampey et al., 2016). The authors showed interaction of synthetic TAR small RNAs and TLRs using pull down assays, however, we note that it is not currently known how RNAs entering the cell naturally through EVs access the endosomal TLRs. Another study showed that HIV‐encoded miRNAs derived from the viral long terminal repeat region, vmiR88 and vmiR99 are transferred in EVs and stimulate TNFα release from recipient macrophages through TLR8 signaling (Barclay et al., 2017; Bernard et al., 2014; Sampey et al., 2016). These RNA‐induced functions are proposed to contribute to the chronic immune activation seen in HIV‐1 infected patients (Barclay et al., 2017; Bernard et al., 2014; Sampey et al., 2016).

It has also been reported in other contexts including cancer studies that small RNAs contained in EVs can interact with TLRs (Driedonks & Nolte‐'t Hoen, 2018; Fabbri, Paone, Calore, Galli, & Croce, 2013; van der Grein & Nolte‐'t Hoen, 2014). Further work is required to understand how RNAs access TLRs in the endosomal system, for example, whether the RNA is transported across the EV membrane within the endosome, whether the EV is destroyed within the endosome or whether the EV‐RNA is released in the cytoplasm but can subsequently reach the endosome. In general, very little is known about the fate of an EV inside the target recipient cell, and research in this area could advance understanding in multiple biological and disease areas.

### 5.2 Secreted host miRNAs activate immune responses and counteract viral infections

Viral infections not only lead to release of viral small RNAs in EVs but can also alter the extracellular profile of host miRNAs. The functions of extracellular host miRNAs appear to be diverse: they have been proposed to benefit the host by antagonizing viral spreading and replication and in other cases benefit the virus by suppressing antiviral immune responses and facilitating infection (Table 1 and Figure 2).

Several studies have shown that cells infected by viruses release higher levels of specific host miRNAs in EVs. For example, the EVs from HCV‐infected macrophages contain miR‐29 family members which inhibit viral replication in recipient hepatocytes (Y. Zhou et al., 2016). Elevated levels of miR‐29 were also found in EVs isolated from hepatitis B virus (HBV)‐infected hepatocytes (compared to EVs from uninfected hepatocytes). In this case, miR‐29 together with miR‐21 was transferred from hepatocytes to recipient macrophages. The transfer of both miRNAs resulted in suppression of the innate immune response by inhibiting IL‐12 expression, a cytokine known to activate natural killer cells (Kouwaki et al., 2016). These two examples demonstrate how the same extracellular miRNA, miR‐29, might have different functions (antiviral vs. proviral) in different contexts. Understanding the natural recipient/target cell of the EVs is imperative to understanding the function of the EV RNA cargo.

Another interesting example showing the functional relevance of extracellular miRNAs comes from the chromosome 19 miRNA cluster (C19MC), which is found in EVs derived from human placental trophoblasts (Delorme‐Axford et al., 2013). This miRNA cluster is almost exclusively expressed in the placenta (Bentwich et al., 2005) and represents the most abundant miRNAs in trophoblastic EVs (Donker et al., 2012). When transferred to nonplacental cells these miRNAs induce upregulation of pro‐autophagy transcripts which inhibit viral replication of several viruses (Figure 2), including poliovirus, vaccinia virus, and herpes simplex virus 1 (Delorme‐Axford et al., 2013). However, a different study suggests that the antiviral effect of the EVs might be at least partly mediated by the unique protein and phospholipid composition (Ouyang et al., 2016). These examples illustrate a challenge in the EV field: how to delineate the importance and function of the RNA from the rest of the EV cargo (Box 1).

BOX 1 **THE BIG QUESTIONS AND CHALLENGES IN EXTRACELLULAR RNA RESEARCH**

1

**Table:** 

**Separating RNA signals from waste** Do the same cells release RNA both as a signaling mechanism and a clearance mechanism? Do these involve different ribonucleoprotein complexes of EV subsets? Technological developments are required to separate different extracellular RNA‐containing complexes, including different EV subsets. Careful consideration is required to prevent contaminants, for example, from calf serum used in cell culture experiments. **Linking intracellular and extracellular RNA regulation** Does the up‐ or down‐regulation of RNAs upon infection inside the cell influence extracellular RNA levels? Do passive or active sorting mechanisms dictate what is released? Have viruses evolved to control these processes? Basic research is required to identify the factors required for RNA sorting/export and genetic and synthetic strategies are needed to block these processes for functional interrogation. **Concentration and localization of imported RNA** Is the amount of RNA imported into a cell is sufficient to raise its concentration to a functional level, in particular for RNAs involved in gene silencing? Quantitative data on imported RNA levels under physiological conditions are required, along with new methods to track imported RNAs, including studies of cross‐species RNA transfer. Further understanding on the subcellular localization of imported RNA is required to understand the relationship between its concentration and activity. **Vesicular RNA: When the whole is greater than the sum of the parts** Is RNA the only functional cargo of a given EV population? Does RNA work in concert with the other signaling molecules? How do we interrogate the contribution of the RNA cargo? Further characterization of the complexes in EVs (ribonucleoprotein complexes) will enable more sophisticated reductionist approaches to study extracellular RNA. Strategies to block imported RNA in recipient cells (e.g. synthetic inhibitors) will help distinguish the functions of RNAs from other EV cargos.

### 5.3 Secreted host miRNAs promote viral infection and contribute to pathology

Several studies have demonstrated that host miRNAs secreted in EVs can also promote infection (Table 1), and it is tempting to speculate that viruses might have evolved mechanisms to modulate the sorting of extracellular miRNAs. EVs derived from EV71‐infected cells, for example, were preferentially enriched for miR‐146a, the miRNA that has been well‐documented to inhibit type I IFN responses (as described above). It was shown that the transfer of this miRNA in EVs resulted in suppression of type I IFN responses in recipient cells (Fu et al., 2017). In this report, the EVs were also found to contain the RISC proteins Argonaute 2 (AGO2) and GW182 and the authors suggest the transfer of the miRNA‐RISC complex might enable suppression of targets immediately after cell entry. miR‐146a was also reported to be strongly increased in EVs isolated from cells coinfected with reticuloendotheliosis virus and avian leukosis virus subgroup J. Although a function of the secreted miRNAs was not explicitly shown in this report, bioinformatic target prediction suggests participation in virus–vector integration and energy metabolism (D. Zhou et al., 2018). Since miR‐146 is known to be upregulated inside infected cells, it will be of interest to understand if the increased secretion of miR‐146 involves passive or active sorting mechanisms (Box 1).

Another report demonstrates increased levels of several miRNAs, including miR‐17‐5p, in EVs isolated from lung epithelial cells and bronchoalveolar lavage fluid of Influenza A virus (IAV)‐infected patients. Transfer of this miRNA to primary alveolar cells leads to the downregulation of the antiviral factor MX1, thereby potentiating IAV infection (Scheller et al., 2018). EV‐miRNAs also contribute to the pathogenesis of the Japanese Encephalitis virus infection, in this case let‐7a/b secretion is increased upon infection of microglia cells and these miRNAs are transferred to neurons, causing damage and inflammation (Mukherjee et al., 2018). Infection can also lead to an increase in secretion of miRNAs that are thought to be antiviral. In theory, this could involve viral mechanisms aimed at reducing miRNA levels inside the infected cell, or host mechanisms to transfer antiviral signaling to uninfected cells. For example, there is some evidence that HIV‐1 protein Nef mediates the selective export of miRNAs which have been predicted to target the viral genome or activate antiviral immune responses (Aqil et al., 2014). Given the diversity of miRNA functions inside of infected cells, it is expected that many more examples will emerge for their functions in transmitting information to uninfected cells, which may also fill‐in some gaps in knowledge of viral‐induced pathologies.

### 5.4 Impact of extracellular RNAs on virus‐induced pathologies: Cancer and fibrosis

Emerging data suggest EVs and miRNAs contribute to viral‐induced malignant transformation of cells infected with KSHV, a gamma herpesvirus associated with abnormal endothelial proliferation and oncogenesis. During latent infection, only a small subset of genes, including 12 viral miRNAs, are expressed. A recent study showed these viral miRNAs are transmitted in EVs from infected to adjacent, healthy cells, where they induce a metabolic shift toward aerobic glycolysis. The viral miRNAs act on targets that reduce mitochondrial respiration in the recipient cells. This shift to aerobic glycolysis is characteristic of cancer cells and establishes a novel mechanism by which viral miRNAs can manipulate the tumor microenvironment (Yogev et al., 2017).

The transmission of cellular miRNAs from virus‐infected cells has also been associated with liver fibrosis. Chronic HCV infection is associated with high risk of liver fibrosis and primary hepatocellular carcinoma (Affo, Yu, & Schwabe, 2017). Hepatic stellate cells are liver‐specific mesenchymal cells regarded as major players in tumor initiation and development and are a major source of extracellular proteins during fibrogenesis (Thompson, Conroy, & Henderson, 2015; Yin, Evason, Asahina, & Stainier, 2013). While HCV cannot infect stellate cells, Devhare et al. showed that virally infected hepatocytes secret EVs that contain miRNAs capable of activating genes related to liver fibrosis in the stellate cells, by targeting and suppressing their inhibitors. Among the secreted miRNAs is miR‐19a, a fibrosis enhancer that targets SOC3, a powerful negative regulator of STAT3 signaling associated with liver inflammation, fibrosis and carcinogenesis (G. He & Karin, 2011; Devhare et al., 2017). Together, these examples demonstrate the impact that extracellular miRNAs from infected cells can have on the microenvironment and how these molecules can shape not only the outcome of the viral infection but also associated pathologies.

## 6 OTHER FUNCTIONS OF EXTRACELLULAR RNA IN VIRAL INFECTIONS

### 6.1 Transmission of HCV in EVs—A role for miR‐122?

**Figure 3:**
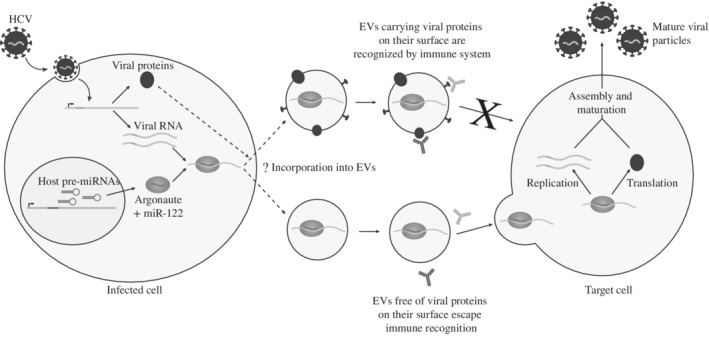
Proposed mechanism for transmission of hepatitis C virus (HCV) in extracellular vesicles (EVs) in complex with miR‐122. HCV genomic RNA is exported from infected cells in EVs in a complex with miR‐122 and Argonaute 2 (AGO2) protein. The absence of viral surface protein prevents immune recognition and allows the transfer to uninfected cells where the virus replicates. EVs harboring viral proteins on their surface are recognized by the immune system and fail to establish viral infection in uninfected cells

In the liver miRNome, miR‐122 accounts for more than 50% of total miRNA. This miRNA is involved in normal liver development and homeostasis and is an essential host‐factor required for efficient HCV replication (Shimakami et al., 2012). A recent study showed that HCV‐infected hepatocytes release EVs that contain complexes of viral RNA, AGO2 and miR‐122 both in vivo and in hepatoma cell lines (Bukong et al., 2014). It has been shown that EVs collected from HCV‐infected cells can initiate in vitro a productive infection (Figure 3), even when treated with antibodies isolated from chronic HCV patients (Bukong et al., 2014; Ramakrishnaiah et al., 2013). However, other reports found that EVs derived from infected cells carry on their surface viral glycoproteins able to trigger immune responses (Masciopinto et al., 2004). These examples highlight one issue in the EV field: there may be multiple EV subsets that derive from an individual cell, that behave differently (Box 1). One report by Dreux et al. (2012) showed that the HCV RNA cargo of EVs from infected cells activates plasmacytoid dendritic cells, which could be a host strategy to elicit an innate immune response in nonpermissive cells. Alternatively, the export of HCV RNA could be a viral strategy to evade detection of the RNA in the infected cell. As with many viral–host interactions, it is not trivial to understand who is calling the shots and further understanding of how RNA export is controlled may shed light on these issues.

What is the importance of the miR‐122‐HCV interaction in EVs—Does this play any role in localization, export and transmission of the virus, beyond stabilization of the viral genome? Further work is required to understand the mechanisms involved here and this example underscores the concept that we should not limit our thinking on the function of miRNAs in viral infection to just the infected cell, or to canonical miRNA functions in gene silencing.

### 6.2 HBV extracellular particles—A secret shuttle for RNA?

During its replicative cycle, HBV is known to produce excess hepatitis B surface proteins (HBs) (Heermann et al., 1984), viral proteins that can self‐assemble into empty, subviral particles that lack a genome (Berkower et al., 2011) but can bind to cell surface receptors (Chai et al., 2008). A recent study showed that HBs particles isolated from peripheral blood of HBV‐infected individuals contain miRNAs and AGO2. In particular, the HBs particles carry liver‐specific miRNAs, such as miR‐122, as well as immune regulatory miRNAs including miR‐223 and miR‐106b. The authors suggest that most of these miRNA target genes associated with viral transmission, including clathrin‐mediated endocytosis and virus entry. This raises the question of whether HBs particles play a role in viral spread and pathogenesis through their miRNA cargo and how miRNA incorporation into HBs particles is dictated. This study found HBs‐associated miRNAs to be different from EV‐miRNAs released from infected cells, which could indicate distinct mechanisms of miRNA sorting and secretion (Novellino et al., 2012). These results were confirmed by another study that was able to isolate miRNAs from extracellular HBs particles (W. Wang et al., 2016).

## 7 LOOKING BEYOND miRNAs—RIBOSOMAL RNA AND TRANSFER RNA FRAGMENTS IN EXTRA‐CELLULAR ENVIRONMENTS

The majority of literature on extracellular RNAs focuses on miRNAs and this bias is most likely due to the fact that miRNAs are relatively easy to identify and there is a model for how they function. However, there is a mounting consensus in the literature suggesting miRNAs are not the most abundant RNA species in EVs. Rather, fragments of other noncoding RNAs including ribosomal RNA (rRNA), transfer RNA (tRNA) YRNAs, 7S RNA, and Vault RNAs are generally more abundant in EVs than individual miRNAs and their levels in EVs are enriched compared to intracellularly (Chahar, Corsello, Kudlicki, Komaravelli, & Casola, 2018; Driedonks et al., 2018; Lässer et al., 2017; Nolte‐'t Hoen et al., 2012; Pérez‐Boza, Lion, & Struman, 2018; Tosar et al., 2015; Wei et al., 2017). Although fragmented noncoding RNAs have historically been considered degradation products, in many cases the fragments arise from specific processing events. Fragments from tRNAs (tRFs) in particular are a ubiquitous class of small RNAs reported across diverse organisms both intracellularly and extracellularly. The fragments are divided into subtypes depending on their biogenesis which can be mediated by Dicer and other RNases including angiogenin (S. Li, Xu, & Sheng, 2018). In 2009, one report suggested that tRFs are involved in regulating cell proliferation in mammalian cells (Y. S. Lee, Shibata, Malhotra, & Dutta, 2009) and several reports since have linked tRF levels inside of cells to viral infection. For example, it has been shown that tRNA‐halves are enriched intracellularly in different viral infections (Jackowiak et al., 2017; Selitsky et al., 2015; Q. Wang et al., 2013) and might promote RSV replication by repressing the mRNA of an antiviral host protein in the cytoplasm (Deng et al., 2015; Q. Wang et al., 2013). There are two examples that viral infections also affect the secretion of tRFs. Zhang et al. (2014) demonstrated increased levels of tRFs in the serum of patients infected with HBV but did not report a function of the tRFs. Another study reported that tRF‐3019, derived from the 3′ end of tRNA‐proline, was detected in the supernatants of cells infected with human T‐cell leukemia virus type 1 (HTLV‐1). There is some evidence that this tRF might enhance viral replication by functioning as a primer for HTLV‐1 reverse transcriptase (Ruggero et al., 2014). Although there is still not one specific model for how and why tRNA fragments function in extracellular signaling processes, they have also recently been linked to immune activation as there are preferentially exported in EVs from T cells and regulate T cell activation (Chiou, Kageyama, & Ansel, 2018). Furthermore, tRNA and rRNA fragments are produced by a broad range of organisms and have been indicated to be important in the context of other parasite–host and microbial–host interactions (X. He et al., 2018; Lambertz et al., 2015).

Further progress in understanding the functions of these noncoding RNA fragments will require modifications to current technical and bioinformatics methodologies. For example, rRNA‐derived fragments (rRFs) are either depleted during library preparation or discarded in small RNA‐seq analysis (Kopylova, Noé, & Touzet, 2012; Schmieder, Lim, & Edwards, 2012; Y. Wang, Li, Sun, & Yao, 2016). This makes it difficult to estimate the proportion of rRFs in EVs. However, a recent study on EVs from human glioma stem cells has found that even in rRNA‐depleted libraries these rRNA fragments make up to one third of the reads, indicating that rRNAs are a dominant component of some EVs (Wei et al., 2017). Only recently, scientists have started to investigate in more detail rRNAs inside the cell and found some evidence for specific processing of rRNA fragments in plants, ticks, and humans (Asha & Soniya, 2017; Z. Chen et al., 2017; Y. Wang, et al., 2016). Although tRFs have been reported as a dominant class of RNA in EVs (Nolte‐'t Hoen et al., 2012), these are also enriched in the nonvesicular fractions of body fluids and cell culture media, which raises the question of how these can be protected from degradation (Ruggero et al., 2014; Tosar et al., 2015, 2018; Zhang et al., 2014). There is some evidence that the tRFs are bound to ribonucleoproteins (Tosar et al., 2015) and they have also been recently reported to exist as stable dimers (Tosar et al., 2018).

Further work on the functions of rRNA and tRNA fragments inside the cells will help create a framework for why and how specific sequences are exported and whether this could be used by viruses or the host defense system. Assuming the export of certain small RNA species from a cell has an important functional role, it is expected that this process would be controlled, and perhaps targeted, by the virus. There is still little understanding on small RNA export and uptake mechanisms but this is an area of active investigation.

## 8 RNA EXPORT MECHANISMS

**Figure 4:**
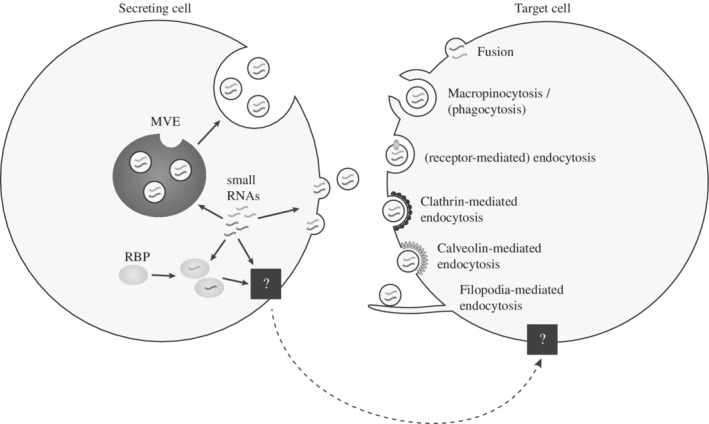
Release and uptake mechanisms of RNA via extracellular vesicles (EVs). EVs containing RNAs can be released from cells either through budding from the plasma membrane or through endosomal pathways involving the multivesicular endosomes (MVEs). Uptake mechanisms in the target cells include fusion with the plasma membrane, phagocytosis or macropinocytosis, (receptor‐mediated) endocytosis, clathrin‐dependent endocytosis, caveolin‐mediated endocytosis, and endocytosis involving filopodia. The mechanism for secretion of small RNAs independent of EVs but in association with RNA‐binding proteins (RBPs) is still unknown (black box)

Extracellular RNA is found outside of cells either protected in EVs or bound to proteins including AGO or high density lipoproteins (Fritz et al., 2016; Sohel, 2016). Therefore, it is generally expected that RNA can be released from cells in association with different complexes, yet the only export mechanism that is understood in any detail is the release of EVs (Figure 4). As described above, the main classes of EVs originate from plasma membranes or the endocytic pathway and both have been shown to contain RNA (Crescitelli et al., 2013). Comparative RNA profiling studies revealed that intracellular and extracellular RNA content is different in some cell types and contexts (Mateescu et al., 2017) leading to interest in what dictates selectivity. Several studies support a model of selective loading of miRNAs into EVs based on sequence recognition by RNA binding proteins, including motifs within the mature sequence such as the EXOmotif GGAG sequence in miR‐601 that is recognized by the heterogeneous nuclear ribonucleoprotein A2B1 (hnRNPA2B1) in T cells (Villarroya‐Beltri et al., 2013) or the GGCU/A motif recognized by the RNA‐binding protein (RBP) SYNCRIP in hepatocytes (Hobor et al., 2018; Santangelo et al., 2016). It has also been shown that miRNAs in EVs show enrichment for nontemplated 3′ end uridylation, compared to cellular miRNAs, but it is not yet known whether this modification represents an active sorting mechanism (Koppers‐Lalic et al., 2014). In addition, the AGO proteins may play a role in miRNA export under certain conditions as phosphorylation of AGO2 prevents its packaging into EVs and this has been shown to influences the levels of specific miRNAs in EVs (McKenzie et al., 2016).

## 9 UPTAKE MECHANISMS OF EV‐RNA: COMMONALITIES WITH VIRUSES

Just as there is a variety of export vehicles for extracellular RNA that includes EVs, RBPs and lipoproteins, it is expected that there are different routes to cross the plasma membrane (Figure 4). However, all of the literature on RNA import into mammalian cells has focused on uptake of EVs. In this context, since EVs and viruses both deliver bioactive material to target cells it is not surprising that there are many overlaps in the pathways involved in export and uptake. Similar to viral tropism, targeted cell uptake of EV‐associated extracellular RNA may be dependent on specific interactions between receptors/recognition molecules on the plasma membrane and adhesion molecules enriched on the surface of EVs. A vast array of proteins, lipids and glycans have been implicated in EV‐cell target interactions including tetraspanins, integrins, extracellular matrix components, immunoglobulins, proteoglycans, and lectins (French, Antonyak, & Cerione, 2017; Mulcahy, Pink, & Carter, 2014; Schorey, Cheng, Singh, & Smith, 2015; van Dongen et al., 2016). There is intriguing data to suggest integrins in particular enable tumor‐derived EVs to target‐specific recipient cells within a tissue, thereby providing a mechanism of priming specific organs for metastatic invasion (Hoshino et al., 2015). To date, it is not known whether different repertoires of adhesion molecules present on EV subpopulations (viral or host) lead to selective uptake by particular cells during infection, but given the precedent in cancer studies, it is certainly possible.

After target cell binding, internalization of EV cargo can be achieved by direct fusion with the plasma membrane (Del Conde, Shrimpton, Thiagarajan, & López, 2005; Montecalvo et al., 2012; Parolini et al., 2009) or via endocytic pathways (Montecalvo et al., 2012; Morelli et al., 2004; Svensson et al., 2013; Tian et al., 2014). Live cell imaging studies in human melanoma cells using EVs labeled with a general lipophilic dye (R18) showed that at least some vesicles are able to fuse with recipient cells and this was enhanced at acidic pHs (Parolini et al., 2009). There are a number of examples of viruses exploiting direct fusion with the plasma membrane for uptake of their genomes (van Dongen et al., 2016). Direct fusion might provide distinct pathways for RNA transfer and function compared to endocytic pathways. At the same time, experimental evidence across many cell types suggests endocytosis is a major route of uptake of both EVs (Costa Verdera, Gitz‐Francois, Schiffelers, & Vader, 2017) and viruses (Mercer, Schelhaas, & Helenius, 2010) while phagocytosis may be used by specialized immune cells (e.g., DCs and macrophages) to internalize EVs (Feng et al., 2010; Rudt & Müller, 1993). Internalization can also occur via macropinocytosis (Fitzner et al., 2011; Nakase, Kobayashi, Takatani‐Nakase, & Yoshida, 2015), clathrin‐dependent (Escrevente, Keller, Altevogt, & Costa, 2011; Tian et al., 2014) as well as clathrin‐independent mechanisms (Costa Verdera et al., 2017) or caveolin‐dependent endocytosis, which involves internalization of specialized micro‐domains called rafts (lipid or caveolae) (Nanbo, Kawanishi, Yoshida, & Yoshiyama, 2013). One recent paper demonstrated that single EVs are recruited to cell bodies by surfing on filopodia that enable them to reach endocytic hot spots (Heusermann et al., 2016). This strategy is also used by viruses, which may have actually evolved to exploit these components of EV uptake pathways. This work went on to trace the subcellular fate of EVs inside of cells, providing evidence that they associate with the ER, a site shown to be important for potent RNA silencing of miRNAs and siRNAs (Li et al., 2013; Stalder et al., 2013).

## 10 METHODS FOR VISUALIZATION OF EVs AND TRACKING OF FUNCTIONAL EXTRACELLULAR RNA TRANSPORT IN VIVO

To date the above example is one of only a few reports providing information on the subcellular localization of EVs following internalization in recipient cells. Advances in single molecular imaging technologies and EV labeling strategies will continue to be important to advance the field, as well as studies under physiologically relevant conditions. Novel mouse transgenic approaches have recently provided compelling evidence for transfer of RNA between via EVs in vivo. The Cre‐loxP transgenic mouse system was used by Stefan Momma's group to show direct transfer of EVs containing Cre mRNA from the blood to the brain in response to inflammation (Ridder et al., 2014). In this model, the Cre recombinase is specifically expressed in hematopoietic cells which were shown to release EVs containing Cre recombinase mRNA (Ridder et al., 2014, 2015) that were taken up by target cells and led to the expression of a Cre‐inducible reporter gene. Although the efficiency of recombination in neurons was low under normal condition, there was an increase in response to inflammation which may indicate that EV‐mediated transfer is important during certain disease states. This approach has also been recently used to visualize EV transfer between cancer cells using a color switch assay, where DsRed+ tumor cells are converted to eGFP+ tumor cells upon uptake of tumor EVs contain CRE mRNA (Zomer et al., 2015; Zomer, Steenbeek, Maynard, & van Rheenen, 2016). The universal use of the Cre‐loxP system/reporter system and the possibility that other cell types may also use EV‐mediated transfer of Cre mRNA offers new avenues to study how EV uptake is mediated and controlled in vivo. However, there is a need for better tools to visualize small RNA trafficking in vivo, and there is no literature to date visualizing their movement in an organism under physiological conditions. In the absence of tools to study small RNA transfer within an organism, studies of cross‐species RNA transfer may provide novel insights to the field. Here, the transferred RNA can be distinguished through sequencing. For example, using a humanized transgenic mouse model expressing the entire primate‐specific C19MC miRNA cluster or individual miRNAs in the placenta, the Sadovsky group where able to show extracellular RNA transport between maternal and fetal compartments in vivo (Chang et al., 2017). It could be envisaged that such a model could be adapted to shed light on the role of this locus in viral infection when ectopically expressed in nonplacenta cells (see Section 5.2).

## 11 CHALLENGES WHEN STUDYING EXTRACELLULAR RNA IN VIRAL INFECTIONS

A key hurdle in the field is purifying sufficient quantities and homogenous populations of EVs for functional studies. Many strategies are now focused on this aspect, which is reviewed further in Konoshenko et al. (2018) In particular, immunopurification strategies targeting surface EV proteins and cushioned density gradient ultracentrifugation (cushioned‐DGUC) techniques are gaining popularity (K. Li, Wong, Hong, & Raffai et al., 2018). Other interesting and novel approaches include Contact Free‐Sorting using acoustic waves to fractionate EVs based on size and density (K. Lee, Shao, Weissleder, & Lee, 2015; M. Wu et al., 2017). Isolating EVs from cell culture media also comes with a range of challenges and extensive energy from the community has focused on “best practice” to avoid EV damage and artifacts associated with the purification methods. An overview of guidelines, advantages and disadvantages of purification approaches is provided in the minimal information for studies of extracellular vesicles 2018 report (Théry et al., 2018). However, this is further complicated when isolating EVs from cells infected with viruses since EVs and virions display similar sizes and biochemical compositions such that commonly used isolation methods will result in a mixed population of EVs and virions (Raab‐Traub & Dittmer, 2017). Affinity purification using magnetic beads against specific EV membrane proteins like CD63 can help separate EVs from some virions but selects for a subpopulation of EVs, as not all EVs express the same markers on their surface (Kowal et al., 2016). This becomes further complicated by the fact that several viruses use the same host secretion routes as vesicles. For example, IAV was shown to incorporate CD9 into virions (Hutchinson et al., 2014), while HBV envelope proteins colocalize with MVE proteins ALIX and VPS4B (Watanabe et al., 2007). Other host proteins that are used as EV markers, including Annexin 2 and heat shock proteins, were found in HIV‐1 virions (Linde et al., 2013). Early studies have shown that even CD63, a classical EV marker, is incorporated by HIV‐1 virions (Meerloo et al., 1993; Orentas & Hildreth, 1993) and this further underscores the difficulty of finding a universal marker to separate EVs from viruses. Accordingly, it is important to note that not only EV purifications are contaminated by viruses but also vice versa, as most virus purification protocols do not deplete EVs, something that has been recognized for many years in the HIV field (Bess, Gorelick, Bosche, Henderson, & Arthur, 1997; Gluschankof, Mondor, Gelderblom, & Sattentau, 1997). Dettenhofer & Yu (1999) were the first to address this issue by developing an OptiPrep velocity gradient to separate HIV particles from EVs in order to characterize the protein profile of the virions. On this note, a range of studies have characterized cellular noncoding RNAs in virions, including small RNAs (Eckwahl, Telesnitsky, & Wolin, 2016) and further work may be required to understand whether these are solely packaged in virions or the EVs with which they co‐purify.

Another major issue in EV purifications is contaminants from the culture media in the case of in vitro studies (Tosar et al., 2017) or from different body fluids in the case of in vivo studies (Gyorgy et al., 2011; Théry, Amigorena, Raposo, & Clayton, 2006). Many cells depend on culture media containing animal‐derived serum, including fetal bovine serum, which is known to comprise bovine EVs containing RNA, as well as nonvesicular complexes containing small RNAs (Tosar et al., 2017). To study EVs and/or extracellular RNA, the standard technique is to use vesicle‐depleted media. Vesicle depletion is usually achieved by prolonged ultracentrifugation of the diluted serum (Shelke, Lässer, Gho, & Lötvall, 2014; Théry et al., 2006) but it has been shown that this is not sufficient to remove EVs completely (Lehrich, Liang, Khosravi, Federoff, & Fiandaca, 2018; Wei, Batagov, Carter, & Krichevsky, 2016). In addition, the depletion of vesicles from the culture media can alter cell proliferation (Eitan, Zhang, Witwer, & Mattson, 2015). Given the importance and breadth of the extracellular RNA field, there is substantial energy focused on addressing these issues and bringing new tools to this research (Carpintero‐Fernández, Fafián‐Labora, & O'Loghlen, 2017; Driedonks, Nijen Twilhaar, & Nolte‐'t Hoen, 2019).

## 12 OUTSTANDING CHALLENGES AND CONCLUSIONS

In summary, the field of extracellular RNA offers many new exciting possibilities for how cells communicate with one another, and how viruses have evolved to exploit or modulate these mechanisms during their lifecycles. However, there are many technical challenges and unknowns that come with the youth of this field (Box 1). Sequencing analyses of the RNA found in different body fluids have dominated this field, with less focus on how the RNA became extracellular and whether it is directed to a specific target. As such, the field of extracellular RNA has lurked on the fringe of mainstream RNA biology but recent reports linking selectivity in RNA export with RBPs will hopefully help to integrate extracellular and intracellular studies. Furthermore, the study of how viruses modulate and hijack RNA export pathways could play an important role in driving the extracellular RNA field forward, in the same way viruses have helped illuminate fundamental aspects of RNA processing and gene regulation inside the cell.

However, many technical challenges exist that need to be addressed to move the field forward. In particular, the ability to study RNA export, including selectivity mechanisms, requires isolation of pure populations of extracellular RNA. We are still learning what those populations are and they include both vesicular and nonvesicular complexes. In the case of vesicular RNAs, we know the EVs themselves are heterogenous: cells produce multiple EV subsets whose cargo and functions can differ (Kowal et al., 2016). In the context of an infected cell, virions themselves also add to this complexity as most current methods of EV or virus purification will not distinguish the two. To meet these challenges, new technologies are evolving, as detailed in this review. Given the burgeoning body of data detailing functional properties of extracellular vesicles in mammalian disease, and the implications that RNA underpins some of these functions, it seems worth the challenge.

## CONFLICT OF INTEREST

The authors have declared no conflicts of interest for this article.

## RELATED WIREs ARTICLE

RNA in extracellular vesicles

## References

[wrna1535-bib-0001] Affo, S. , Yu, L.‐X. , & Schwabe, R. F. (2017). The role of cancer‐associated fibroblasts and fibrosis in liver cancer. Annual Review of Pathology, 12, 153–186. 10.1146/annurev-pathol-052016-100322 PMC5720358 27959632

[wrna1535-bib-0002] Alvarez‐Garcia, I. , & Miska, E. A. (2005). MicroRNA functions in animal development and human disease. Development, 132(21), 4653–4662. 10.1242/dev.02073 16224045

[wrna1535-bib-0003] Aqil, M. , Naqvi, A. R. , Mallik, S. , Bandyopadhyay, S. , Maulik, U. , & Jameel, S. (2014). The HIV Nef protein modulates cellular and exosomal miRNA profiles in human monocytic cells. Journal of Extracellular Vesicles, 3, 23129 10.3402/jev.v3.23129 PMC3967016 24678387

[wrna1535-bib-0004] Archer, S. J. (1978). Induction of a T‐cell specific antigen on bone marrow lymphocytes with thymus RNA. Immunology, 34, 123–129. 75176 PMC1457351

[wrna1535-bib-0005] Asha, S. , & Soniya, E. V. (2017). The sRNAome mining revealed existence of unique signature small RNAs derived from 5.8SrRNA from *Piper nigrum* and other plant lineages. Scientific Reports, 7, 41052 10.1038/srep41052 28145468 PMC5286533

[wrna1535-bib-0006] Azlan, A. , Dzaki, N. , & Azzam, G. (2016). Argonaute: The executor of small RNA function. Journal of Genetics and Genomics, 43(8), 481–494. 10.1016/j.jgg.2016.06.002 27569398

[wrna1535-bib-0007] Backes, S. , Shapiro, J. S. , Sabin, L. R. , Pham, A. M. , Reyes, I. , Moss, B. , … tenOever, B. R. (2012). Degradation of host microRNAs by poxvirus poly(A) polymerase reveals terminal RNA methylation as a protective antiviral mechanism. Cell Host & Microbe, 12, 200–210. 10.1016/J.CHOM.2012.05.019 22901540 PMC3782087

[wrna1535-bib-0008] Baglio, S. R. , van Eijndhoven, M. A. J. , Koppers‐Lalic, D. , Berenguer, J. , Lougheed, S. M. , Gibbs, S. , … Pegtel, D. M. (2016). Sensing of latent EBV infection through exosomal transfer of 5′pppRNA. Proceedings of the National Academy of Sciences of the United States of America, 113, E587–E596. 10.1073/pnas.1518130113 26768848 PMC4747727

[wrna1535-bib-0009] Barclay, R. A. , Schwab, A. , DeMarino, C. , Akpamagbo, Y. , Lepene, B. , Kassaye, S. , … Kashanchi, F. (2017). Exosomes from uninfected cells activate transcription of latent HIV‐1. Journal of Biological Chemistry, 292, 14764 10.1074/jbc.A117.793521 28887434 PMC5592657

[wrna1535-bib-0010] Bartel, D. P. (2018). Metazoan microRNAs. Cell, 173, 20–51. 10.1016/j.cell.2018.03.006 29570994 PMC6091663

[wrna1535-bib-0011] Baulcombe, D. (2004). RNA silencing in plants. Nature, 431, 356–363. 10.1038/nature02874 15372043

[wrna1535-bib-0012] Becker, A. , Thakur, B. K. , Weiss, J. M. , Kim, H. S. , Peinado, H. , & Lyden, D. (2016). Extracellular vesicles in cancer: Cell‐to‐cell mediators of metastasis. Cancer Cell, 30, 836–848. 10.1016/J.CCELL.2016.10.009 27960084 PMC5157696

[wrna1535-bib-0013] Bentwich, I. , Avniel, A. , Karov, Y. , Aharonov, R. , Gilad, S. , Barad, O. , … Bentwich, Z. (2005). Identification of hundreds of conserved and nonconserved human microRNAs. Nature Genetics, 37, 766–770. 10.1038/ng1590 15965474

[wrna1535-bib-0014] Berkower, I. , Spadaccini, A. , Chen, H. , Al‐Awadi, D. , Muller, J. , Gao, Y. , … Ni, Y. (2011). Hepatitis B virus surface antigen assembly function persists when entire transmembrane domains 1 and 3 are replaced by a heterologous transmembrane sequence. Journal of Virology, 85, 2439–2448. 10.1128/JVI.02061-10 21177825 PMC3067800

[wrna1535-bib-0015] Bernard, M. A. , Zhao, H. , Yue, S. C. , Anandaiah, A. , Koziel, H. , & Tachado, S. D. (2014). Novel HIV‐1 MiRNAs stimulate TNFα release in human macrophages via TLR8 signaling pathway. PLoS One, 9, e106006 10.1371/journal.pone.0106006 25191859 PMC4156304

[wrna1535-bib-0016] Bess, J. W., Jr. , Gorelick, R. J. , Bosche, W. J. , Henderson, L. E. , & Arthur, L. O. (1997). Microvesicles are a source of contaminating cellular proteins found in purified HIV‐1 preparations. Virology, 230(1), 134–144. 10.1006/viro.1997.8499 9126269

[wrna1535-bib-0017] Brennecke, J. , Stark, A. , Russell, R. B. , & Cohen, S. M. (2005). Principles of microRNA‐target recognition. PLoS Biology, 3(3), e85 10.1371/journal.pbio.0030085 15723116 PMC1043860

[wrna1535-bib-0018] Bruscella, P. , Bottini, S. , Baudesson, C. , Pawlotsky, J.‐M. , Feray, C. , & Trabucchi, M. (2017). Viruses and miRNAs: More friends than foes. Frontiers in Microbiology, 8, 824 10.3389/fmicb.2017.00824 28555130 PMC5430039

[wrna1535-bib-0019] Buck, A. H. , Ivens, A. , Gordon, K. , Craig, N. , Houzelle, A. , Roche, A. , … Stewart, J. P. (2015). Quantitative analysis of microRNAs in Vaccinia virus infection reveals diversity in their susceptibility to modification and suppression. PLoS One, 10(7), e0131787 10.1371/journal.pone.0131787 26161560 PMC4498801

[wrna1535-bib-0020] Bukong, T. N. , Momen‐Heravi, F. , Kodys, K. , Bala, S. , & Szabo, G. (2014). Exosomes from hepatitis C infected patients transmit HCV infection and contain replication competent viral RNA in complex with Ago2‐miR122‐HSP90. PLoS Pathogens, 10, e1004424 10.1371/journal.ppat.1004424 25275643 PMC4183590

[wrna1535-bib-0021] Carpintero‐Fernández, P. , Fafián‐Labora, J. , & O'Loghlen, A. (2017). Technical advances to study extracellular vesicles. Frontiers in Molecular Biosciences, 4, 79 10.3389/fmolb.2017.00079 29234666 PMC5712308

[wrna1535-bib-0022] Cazalla, D. , Yario, T. , Steitz, J. A. , & Steitz, J. (2010). Down‐regulation of a host microRNA by a herpesvirus saimiri noncoding RNA. Science, 328, 1563–1566. 10.1126/science.1187197 20558719 PMC3075239

[wrna1535-bib-0023] Chahar, H. S. , Corsello, T. , Kudlicki, A. S. , Komaravelli, N. , & Casola, A. (2018). Respiratory syncytial virus infection changes cargo composition of exosome released from airway epithelial cells. Scientific Reports, 8, 387 10.1038/s41598-017-18672-5 29321591 PMC5762922

[wrna1535-bib-0024] Chai, N. , Chang, H. E. , Nicolas, E. , Han, Z. , Jarnik, M. , & Taylor, J. (2008). Properties of subviral particles of hepatitis B virus. Journal of Virology, 82(16), 7812–7817. 10.1128/JVI.00561-08 18524834 PMC2519590

[wrna1535-bib-0025] Chang, G. , Mouillet, J.‐F. , Mishima, T. , Chu, T. , Sadovsky, E. , Coyne, C. B. , … Sadovsky, Y. (2017). Expression and trafficking of placental microRNAs at the feto‐maternal interface. FASEB Journal, 31, 2760–2770. 10.1096/fj.201601146R 28289056 PMC5471515

[wrna1535-bib-0026] Chen, X. , Ba, Y. , Ma, L. , Cai, X. , Yin, Y. , Wang, K. , … Zhang, C.‐Y. (2008). Characterization of microRNAs in serum: A novel class of biomarkers for diagnosis of cancer and other diseases. Cell Research, 18, 997–1006. 10.1038/cr.2008.282 18766170

[wrna1535-bib-0027] Chen, Z. , Sun, Y. , Yang, X. , Wu, Z. , Guo, K. , Niu, X. , … Gao, S. (2017). Two featured series of rRNA‐derived RNA fragments (rRFs) constitute a novel class of small RNAs. PLoS One, 12, e0176458 10.1371/journal.pone.0176458 28441451 PMC5404876

[wrna1535-bib-0028] Chim, S. S. C. , Shing, T. K. F. , Hung, E. C. W. , Leung, T.‐y. , Lau, T.‐k. , Chiu, R. W. K. , & Dennis Lo, Y. M. (2008). Detection and characterization of placental microRNAs in maternal plasma. Clinical Chemistry, 54, 482–490. 10.1373/clinchem.2007.097972 18218722

[wrna1535-bib-0029] Chiou, N. T. , Kageyama, R. , & Ansel, K. M. (2018). Selective export into extracellular vesicles and function of tRNA fragments during T cell activation. Cell Reports, 25(12), 3356–3370. 10.1016/j.celrep.2018.11.073 30566862 PMC6392044

[wrna1535-bib-0030] Chivero, E. T. , Bhattarai, N. , Rydze, R. T. , Winters, M. A. , Holodniy, M. , & Stapleton, J. T. (2014). Human pegivirus RNA is found in multiple blood mononuclear cells in vivo and serum‐derived viral RNA‐containing particles are infectious in vitro. Journal of General Virology, 95, 1307–1319. 10.1099/vir.0.063016-0 24668525 PMC4027039

[wrna1535-bib-0031] Colombo, M. , Raposo, G. , & Théry, C. (2014). Biogenesis, secretion, and intercellular interactions of exosomes and other extracellular vesicles. Annual Review of Cell and Developmental Biology, 30, 255–289. 10.1146/annurev-cellbio-101512-122326 25288114

[wrna1535-bib-0032] Columba Cabezas, S. , & Federico, M. (2013). Sequences within RNA coding for HIV‐1 Gag p17 are efficiently targeted to exosomes. Cellular Microbiology, 15, 412–429. 10.1111/cmi.12046 23072732

[wrna1535-bib-0033] Cosset, F.‐L. , & Dreux, M. (2014). HCV transmission by hepatic exosomes establishes a productive infection. Journal of Hepatology, 60, 674–675. 10.1016/J.JHEP.2013.10.015 24512825

[wrna1535-bib-0034] Costa Verdera, H. , Gitz‐Francois, J. J. , Schiffelers, R. M. , & Vader, P. (2017). Cellular uptake of extracellular vesicles is mediated by clathrin‐independent endocytosis and macropinocytosis. Journal of Controlled Release, 266, 100–108. 10.1016/J.JCONREL.2017.09.019 28919558

[wrna1535-bib-0035] Crescitelli, R. , Lässer, C. , Szabó, T. G. , Kittel, A. , Eldh, M. , Dianzani, I. , … Lötvall, J. (2013). Distinct RNA profiles in subpopulations of extracellular vesicles: Apoptotic bodies, microvesicles and exosomes. Journal of Extracellular Vesicles, 2, 20677 10.3402/jev.v2i0.20677 PMC3823106 24223256

[wrna1535-bib-0036] Cullen, B. R. (2006). Viruses and microRNAs. Nature Genetics, 38, S25–S30. 10.1038/ng1793 16736021

[wrna1535-bib-0037] de Candia, P. , De Rosa, V. , Casiraghi, M. , & Matarese, G. (2016). Extracellular RNAs: A secret arm of immune system regulation. Journal of Biological Chemistry, 291, 7221–7228. 10.1074/jbc.R115.708842 26887954 PMC4817155

[wrna1535-bib-0038] De Toro, J. , Herschlik, L. , Waldner, C. , & Mongini, C. (2015). Emerging roles of exosomes in normal and pathological conditions: New insights for diagnosis and therapeutic applications. Frontiers in Immunology, 6, 203 10.3389/fimmu.2015.00203 25999947 PMC4418172

[wrna1535-bib-0039] Del Conde, I. , Shrimpton, C. N. , Thiagarajan, P. , & López, J. A. (2005). Tissue‐factor‐bearing microvesicles arise from lipid rafts and fuse with activated platelets to initiate coagulation. Blood, 106, 1604–1611. 10.1182/blood-2004-03-1095 15741221

[wrna1535-bib-0040] Delorme‐Axford, E. , Donker, R. B. , Mouillet, J.‐F. , Chu, T. , Bayer, A. , Ouyang, Y. , … Coyne, C. B. (2013). Human placental trophoblasts confer viral resistance to recipient cells. Proceedings of the National Academy of Sciences of the United States of America, 110, 12048–12053. 10.1073/pnas.1304718110 23818581 PMC3718097

[wrna1535-bib-0041] Deng, J. , Ptashkin, R. N. , Chen, Y. , Cheng, Z. , Liu, G. , Phan, T. , … Bao, X. (2015). Respiratory syncytial virus utilizes a tRNA fragment to suppress antiviral responses through a novel targeting mechanism. Molecular Therapy, 23(10), 1622–1629. 10.1038/mt.2015.124 26156244 PMC4817927

[wrna1535-bib-0042] Dettenhofer, M. , & Yu, X. F. (1999). Highly purified human immunodeficiency virus type 1 reveals a virtual absence of Vif in virions. Journal of Virology, 73(2), 1460–1467. 9882352 10.1128/jvi.73.2.1460-1467.1999 PMC103971

[wrna1535-bib-0043] Devhare, P. B. , Sasaki, R. , Shrivastava, S. , Di Bisceglie, A. M. , Ray, R. , & Ray, R. B. (2017). Exosome‐mediated intercellular communication between hepatitis C virus‐infected hepatocytes and hepatic stellate cells. Journal of Virology, 91, e02225–02216. 10.1128/JVI.02225-16 28077652 PMC5331806

[wrna1535-bib-0044] Ding, S.‐W. , Han, Q. , Wang, J. , & Li, W.‐X. (2018). Antiviral RNA interference in mammals. Current Opinion in Immunology, 54, 109–114. 10.1016/J.COI.2018.06.010 30015086 PMC6196099

[wrna1535-bib-0045] Ding, S.‐W. , & Voinnet, O. (2007). Antiviral immunity directed by small RNAs. Cell, 130, 413–426. 10.1016/J.CELL.2007.07.039 17693253 PMC2703654

[wrna1535-bib-0046] Donker, R. B. , Mouillet, J. F. , Chu, T. , Hubel, C. A. , Stolz, D. B. , Morelli, A. E. , & Sadovsky, Y. (2012). The expression profile of C19MC microRNAs in primary human trophoblast cells and exosomes. Molecular Human Reproduction, 18, 417–424. 10.1093/molehr/gas013 22383544 PMC3389496

[wrna1535-bib-0047] Dreux, M. , Garaigorta, U. , Boyd, B. , Décembre, E. , Chung, J. , Whitten‐Bauer, C. , … Chisari, F. V. (2012). Short‐range exosomal transfer of viral RNA from infected cells to plasmacytoid dendritic cells triggers innate immunity. Cell Host & Microbe, 12, 558–570. 10.1016/J.CHOM.2012.08.010 23084922 PMC3479672

[wrna1535-bib-0048] Driedonks, T. A. P. , Nijen Twilhaar, M. K. , & Nolte‐'t Hoen, E. N. M. (2019). Technical approaches to reduce interference of fetal calf serum derived RNA in the analysis of extracellular vesicle RNA from cultured cells. Journal of Extracellular Vesicles, 8(1), 1552059 10.1080/20013078.2018.1552059 30559953 PMC6292350

[wrna1535-bib-0049] Driedonks, T. A. P. , & Nolte‐'t Hoen, E. N. M. (2018). Circulating Y‐RNAs in extracellular vesicles and ribonucleoprotein complexes; implications for the immune system. Frontiers in Immunology, 9, 3164 10.3389/fimmu.2018.03164 30697216 PMC6340977

[wrna1535-bib-0050] Driedonks, T. A. P. , van der Grein, S. G. , Ariyurek, Y. , Buermans, H. P. J. , Jekel, H. , Chow, F. W. N. , … Nolte‐'t Hoen, E. N. M. (2018). Immune stimuli shape the small non‐coding transcriptome of extracellular vesicles released by dendritic cells. Cellular and Molecular Life Sciences, 75(20), 3857–3875. 10.1007/s00018-018-2842-8 29808415 PMC6154026

[wrna1535-bib-0051] Eckwahl, M. J. , Telesnitsky, A. , & Wolin, S. L. (2016). Host RNA packaging by retroviruses: A newly synthesized story. MBio, 7, e02025‐15 10.1128/mBio.02025-15 26861021 PMC4752605

[wrna1535-bib-0052] Eitan, E. , Zhang, S. , Witwer, K. W. , & Mattson, M. P. (2015). Extracellular vesicle‐depleted fetal bovine and human sera have reduced capacity to support cell growth. Journal of Extracellular Vesicles, 4, 26373 10.3402/jev.v4.26373 25819213 PMC4376846

[wrna1535-bib-0053] Escrevente, C. , Keller, S. , Altevogt, P. , & Costa, J. (2011). Interaction and uptake of exosomes by ovarian cancer cells. BMC Cancer, 11, 108 10.1186/1471-2407-11-108 21439085 PMC3072949

[wrna1535-bib-0054] Fabbri, M. , Paone, A. , Calore, F. , Galli, R. , & Croce, C. M. (2013). A new role for microRNAs, as ligands of toll‐like receptors. RNA Biology, 10(2), 169–174. 10.4161/rna.23144 23296026 PMC3594274

[wrna1535-bib-0055] Feng, D. , Zhao, W.‐L. , Ye, Y.‐Y. , Bai, X.‐C. , Liu, R.‐Q. , Chang, L.‐F. , … Sui, S.‐F. (2010). Cellular internalization of exosomes occurs through phagocytosis. Traffic, 11, 675–687. 10.1111/j.1600-0854.2010.01041.x 20136776

[wrna1535-bib-0056] Fishman, M. , Hammerstrom, R. A. , & Bond, V. P. (1963). In vitro transfer of macrophage RNA to lymph node cells. Nature, 198, 549–551. 10.1038/198549a0 13945312

[wrna1535-bib-0057] Fitzner, D. , Schnaars, M. , van Rossum, D. , Krishnamoorthy, G. , Dibaj, P. , Bakhti, M. , … Simons, M. (2011). Selective transfer of exosomes from oligodendrocytes to microglia by macropinocytosis. Journal of Cell Science, 124, 447–458. 10.1242/jcs.074088 21242314

[wrna1535-bib-0058] French, K. C. , Antonyak, M. A. , & Cerione, R. A. (2017). Extracellular vesicle docking at the cellular port: Extracellular vesicle binding and uptake. Seminars in Cell & Developmental Biology, 67, 48–55. 10.1016/J.SEMCDB.2017.01.002 28104520 PMC5484727

[wrna1535-bib-0059] Fritz, J. V. , Heintz‐Buschart, A. , Ghosal, A. , Wampach, L. , Etheridge, A. , Galas, D. , & Wilmes, P. (2016). Sources and functions of extracellular small RNAs in human circulation. Annual Review of Nutrition, 36, 301–336. 10.1146/annurev-nutr-071715-050711 PMC5479634 27215587

[wrna1535-bib-0060] Fruci, D. , Rota, R. , & Gallo, A. (2017). The role of HCMV and HIV‐1 microRNAs: Processing, and mechanisms of action during viral infection. Frontiers in Microbiology, 8, 689 10.3389/fmicb.2017.00689 28484438 PMC5399795

[wrna1535-bib-0061] Fu, Y. , Zhang, L. , Zhang, F. , Tang, T. , Zhou, Q. , Feng, C. , … Wu, Z. (2017). Exosome‐mediated miR‐146a transfer suppresses type I interferon response and facilitates EV71 infection. PLoS Pathogens, 13, e1006611 10.1371/journal.ppat.1006611 28910400 PMC5614653

[wrna1535-bib-0500] Girardi, E ., López, P ., & Pfeffer, S . (2018). On the importance of host microRNAs during viral infection. Frontiers in Genetics, 9, 439 10.3389/fgene.2018.00439 30333857 PMC6176045

[wrna1535-bib-0062] Gluschankof, P. , Mondor, I. , Gelderblom, H. R. , & Sattentau, Q. J. (1997). Cell membrane vesicles are a major contaminant of gradient‐enriched human immunodeficiency virus type‐1 preparations. Virology, 230(1), 125–133. 10.1006/viro.1997.8453 9126268

[wrna1535-bib-0063] Gottwein, E. , & Cullen, B. R. (2010). A human herpesvirus microRNA inhibits p21 expression and attenuates p21‐mediated cell cycle arrest. Journal of Virology, 84, 5229–5237. 10.1128/JVI.00202-10 20219912 PMC2863803

[wrna1535-bib-0064] Gould, S. J. , Booth, A. M. , & Hildreth, J. E. K. (2003). The Trojan exosome hypothesis. Proceedings of the National Academy of Sciences, 100, 10592–10597. 10.1073/pnas.1831413100 PMC196848 12947040

[wrna1535-bib-0065] Grey, F. (2015). Role of microRNAs in herpesvirus latency and persistence. Journal of General Virology, 96, 739–751. 10.1099/vir.0.070862-0 25406174

[wrna1535-bib-0066] Guo, Y. E. , Oei, T. , & Steitz, J. A. (2015). Herpesvirus saimiri microRNAs preferentially target host cell cycle regulators. Journal of Virology, 89, 10901–10911. 10.1128/JVI.01884-15 26292323 PMC4621106

[wrna1535-bib-0067] Guo, Y. E. , Riley, K. J. , Iwasaki, A. , & Steitz, J. A. (2014). Alternative capture of noncoding RNAs or protein‐coding genes by herpesviruses to alter host T cell function. Molecular Cell, 54, 67–79. 10.1016/J.MOLCEL.2014.03.025 24725595 PMC4039351

[wrna1535-bib-0068] Gyorgy, B. , Modos, K. , Pallinger, E. , Paloczi, K. , Pasztoi, M. , Misjak, P. , … Buzas, E. I. (2011). Detection and isolation of cell‐derived microparticles are compromised by protein complexes resulting from shared biophysical parameters. Blood, 117(4), e39–e48. 10.1182/blood-2010-09-307595 21041717

[wrna1535-bib-0069] Ha, M. , & Kim, V. N. (2014). Regulation of microRNA biogenesis. Nature Reviews Molecular Cell Biology, 15, 509–524. 10.1038/nrm3838 25027649

[wrna1535-bib-0070] Hamilton, A. J. , & Baulcombe, D. C. (1999). A species of small antisense RNA in posttranscriptional gene silencing in plants. Science, 286, 950–952. 10542148 10.1126/science.286.5441.950

[wrna1535-bib-0071] Hannon, G. J. (2002). RNA interference. Nature, 418, 244–251. 10.1038/418244a 12110901

[wrna1535-bib-0072] Harwig, A. , Jongejan, A. , van Kampen, A. H. C. , Berkhout, B. , & Das, A. T. (2016). Tat‐dependent production of an HIV‐1 TAR‐encoded miRNA‐like small RNA. Nucleic Acids Research, 44, 4340–4353. 10.1093/nar/gkw167 26984525 PMC4872094

[wrna1535-bib-0073] He, G. , & Karin, M. (2011). NF‐κB and STAT3—Key players in liver inflammation and cancer. Cell Research, 21, 159–168. 10.1038/cr.2010.183 21187858 PMC3193410

[wrna1535-bib-0074] He, X. , Li, F. , Bor, B. , Koyano, K. , Cen, L. , Xiao, X. , … Wong, D. T. W. (2018). Human tRNA‐derived small RNAs modulate host–oral microbial interactions. Journal of Dental Research, 97, 1236–1243. 10.1177/0022034518770605 29702004 PMC6151917

[wrna1535-bib-0075] Heermann, K. H. , Goldmann, U. , Schwartz, W. , Seyffarth, T. , Baumgarten, H. , & Gerlich, W. H. (1984). Large surface proteins of hepatitis B virus containing the pre‐s sequence. Journal of Virology, 52, 396–402. 6492255 10.1128/jvi.52.2.396-402.1984 PMC254539

[wrna1535-bib-0076] Heusermann, W. , Hean, J. , Trojer, D. , Steib, E. , von Bueren, S. , Graff‐Meyer, A. , … Meisner‐Kober, N. C. (2016). Exosomes surf on filopodia to enter cells at endocytic hot spots, traffic within endosomes, and are targeted to the ER. Journal of Cell Biology, 213, 173–184. 10.1083/jcb.201506084 27114500 PMC5084269

[wrna1535-bib-0077] Ho, B.‐C. , Yu, I.‐S. , Lu, L.‐F. , Rudensky, A. , Chen, H.‐Y. , Tsai, C.‐W. , … Yu, S.‐L. (2014). Inhibition of miR‐146a prevents enterovirus‐induced death by restoring the production of type I interferon. Nature Communications, 5, 3344 10.1038/ncomms4344 24561744

[wrna1535-bib-0078] Hobor, F. , Dallmann, A. , Ball, N. J. , Cicchini, C. , Battistelli, C. , Ogrodowicz, R. W. , … Ramos, A. (2018). A cryptic RNA‐binding domain mediates Syncrip recognition and exosomal partitioning of miRNA targets. Nature Communications, 9(1), 831 10.1038/s41467-018-03182-3 PMC5827114 29483512

[wrna1535-bib-0079] Hoshino, A. , Costa‐Silva, B. , Shen, T.‐L. , Rodrigues, G. , Hashimoto, A. , Tesic Mark, M. , … Lyden, D. (2015). Tumour exosome integrins determine organotropic metastasis. Nature, 527, 329–335. 10.1038/nature15756 26524530 PMC4788391

[wrna1535-bib-0080] Hoy, A. M. , & Buck, A. H. (2012). Extracellular small RNAs: What, where, why? Biochemical Society Transactions, 40, 886–890. 10.1042/BST20120019 22817753 PMC3433256

[wrna1535-bib-0081] Hsu, A. C.‐Y. , Dua, K. , Starkey, M. R. , Haw, T.‐J. , Nair, P. M. , Nichol, K. , … Wark, P. A. (2017). MicroRNA‐125a and ‐b inhibit A20 and MAVS to promote inflammation and impair antiviral response in COPD. JCI Insight, 2, e90443 10.1172/jci.insight.90443 28405612 PMC5374076

[wrna1535-bib-0082] Hutchinson, E. C. , Charles, P. D. , Hester, S. S. , Thomas, B. , Trudgian, D. , Martínez‐Alonso, M. , & Fodor, E. (2014). Conserved and host‐specific features of influenza virion architecture. Nature Communications, 5, 4816 10.1038/ncomms5816 PMC4167602 25226414

[wrna1535-bib-0083] Jackowiak, P. , Hojka‐Osinska, A. , Philips, A. , Zmienko, A. , Budzko, L. , Maillard, P. , … Figlerowicz, M. (2017). Small RNA fragments derived from multiple RNA classes—The missing element of multi‐omics characteristics of the hepatitis C virus cell culture model. BMC Genomics, 18, 502 10.1186/s12864-017-3891-3 28666407 PMC5493846

[wrna1535-bib-0084] Jaworski, E. , Narayanan, A. , Van Duyne, R. , Shabbeer‐Meyering, S. , Iordanskiy, S. , Saifuddin, M. , … Kashanchi, F. (2014). Human T‐lymphotropic virus type 1‐infected cells secrete exosomes that contain Tax protein. Journal of Biological Chemistry, 289, 22284–22305. 10.1074/jbc.M114.549659 24939845 PMC4139239

[wrna1535-bib-0085] Jopling, C. L. , Yi, M. , Lancaster, A. M. , Lemon, S. M. , & Sarnow, P. (2005). Modulation of hepatitis C virus RNA abundance by a liver‐specific microRNA. Science, 309, 1577–1581. 10.1126/science.1113329 16141076

[wrna1535-bib-0086] Kincaid, R. P. , & Sullivan, C. S. (2012). Virus‐encoded microRNAs: An overview and a look to the future. PLoS Pathogens, 8, e1003018 10.1371/journal.ppat.1003018 23308061 PMC3534370

[wrna1535-bib-0087] Klase, Z. , Kale, P. , Winograd, R. , Gupta, M. V. , Heydarian, M. , Berro, R. , … Kashanchi, F. (2007). HIV‐1 TAR element is processed by dicer to yield a viral micro‐RNA involved in chromatin remodeling of the viral LTR. BMC Molecular Biology, 8, 63 10.1186/1471-2199-8-63 17663774 PMC1955452

[wrna1535-bib-0088] Klase, Z. , Winograd, R. , Davis, J. , Carpio, L. , Hildreth, R. , Heydarian, M. , … Kashanchi, F. (2009). HIV‐1 TAR miRNA protects against apoptosis by altering cellular gene expression. Retrovirology, 6, 18 10.1186/1742-4690-6-18 19220914 PMC2654423

[wrna1535-bib-0089] Kolodny, G. M. (1971). Evidence for transfer of macromolecular RNA between mammalian cells in culture. Experimental Cell Research, 65, 313–324. 4101797 10.1016/0014-4827(71)90007-3

[wrna1535-bib-0090] Kolodny, G. M. (1972). Cell to cell transfer of RNA into transformed cells. Journal of Cellular Physiology, 79, 147–150. 10.1002/jcp.1040790117 4333154

[wrna1535-bib-0091] Konoshenko, M. Y. , Lekchnov, E. A. , Vlassov, A. V. , & Laktionov, P. P. (2018). Isolation of extracellular vesicles: General methodologies and latest trends. BioMed Research International, 2018, 1–27. 10.1155/2018/8545347 PMC5831698 29662902

[wrna1535-bib-0092] Koppers‐Lalic, D. , Hackenberg, M. , Bijnsdorp, I. V. , van Eijndhoven, M. A. J. , Sadek, P. , Sie, D. , … Pegtel, D. M. (2014). Nontemplated nucleotide additions distinguish the small RNA composition in cells from exosomes. Cell Reports, 8, 1649–1658. 10.1016/J.CELREP.2014.08.027 25242326

[wrna1535-bib-0093] Kopylova, E. , Noé, L. , & Touzet, H. (2012). SortMeRNA: Fast and accurate filtering of ribosomal RNAs in metatranscriptomic data. Bioinformatics, 28, 3211–3217. 10.1093/bioinformatics/bts611 23071270

[wrna1535-bib-0094] Kouwaki, T. , Fukushima, Y. , Daito, T. , Sanada, T. , Yamamoto, N. , Mifsud, E. J. , … Oshiumi, H. (2016). Extracellular vesicles including exosomes regulate innate immune responses to hepatitis B virus infection. Frontiers in Immunology, 7, 335 10.3389/fimmu.2016.00335 27630638 PMC5005343

[wrna1535-bib-0095] Kouwaki, T. , Okamoto, M. , Tsukamoto, H. , Fukushima, Y. , & Oshiumi, H. (2017). Extracellular vesicles deliver host and virus RNA and regulate innate immune response. International Journal of Molecular Sciences, 18, 666 10.3390/ijms18030666 PMC5372678 28335522

[wrna1535-bib-0096] Kowal, J. , Arras, G. , Colombo, M. , Jouve, M. , Morath, J. P. , Primdal‐Bengtson, B. , … Théry, C. (2016). Proteomic comparison defines novel markers to characterize heterogeneous populations of extracellular vesicle subtypes. Proceedings of the National Academy of Sciences, 113, E968–E977. 10.1073/pnas.1521230113 PMC4776515 26858453

[wrna1535-bib-0097] Laganà, A. , Russo, F. , Veneziano, D. , Bella, S. D. , Giugno, R. , Pulvirenti, A. , … Ferro, A. (2013). Extracellular circulating viral microRNAs: Current knowledge and perspectives. Frontiers in Genetics, 4, 120 10.3389/fgene.2013.00120 23805153 PMC3690336

[wrna1535-bib-0098] Lambertz, U. , Oviedo Ovando, M. E. , Vasconcelos, E. , Unrau, P. J. , Myler, P. J. , & Reiner, N. E. (2015). Small RNAs derived from tRNAs and rRNAs are highly enriched in exosomes from both old and new world Leishmania providing evidence for conserved exosomal RNA packaging. BMC Genomics, 16, 151 10.1186/s12864-015-1260-7 25764986 PMC4352550

[wrna1535-bib-0099] Lässer, C. , Shelke, G. V. , Yeri, A. , Kim, D.‐K. , Crescitelli, R. , Raimondo, S. , … Lötvall, J. (2017). Two distinct extracellular RNA signatures released by a single cell type identified by microarray and next‐generation sequencing. RNA Biology, 14, 58–72. 10.1080/15476286.2016.1249092 27791479 PMC5270547

[wrna1535-bib-0100] Lawrie, C. H. , Gal, S. , Dunlop, H. M. , Pushkaran, B. , Liggins, A. P. , Pulford, K. , … Harris, A. L. (2008). Detection of elevated levels of tumour‐associated microRNAs in serum of patients with diffuse large B‐cell lymphoma. British Journal of Haematology, 141, 672–675. 10.1111/j.1365-2141.2008.07077.x 18318758

[wrna1535-bib-0101] Lee, K. , Shao, H. , Weissleder, R. , & Lee, H. (2015). Acoustic purification of extracellular microvesicles. ACS Nano, 9, 2321–2327. 10.1021/nn506538f 25672598 PMC4373978

[wrna1535-bib-0102] Lee, Y. S. , Shibata, Y. , Malhotra, A. , & Dutta, A. (2009). A novel class of small RNAs: tRNA‐derived RNA fragments (tRFs). Genes & Development, 23, 2639–2649. 10.1101/gad.1837609 19933153 PMC2779758

[wrna1535-bib-0103] Lehrich, B. M. , Liang, Y. , Khosravi, P. , Federoff, H. J. , & Fiandaca, M. S. (2018). Fetal bovine serum‐derived extracellular vesicles persist within vesicle‐depleted culture media. International Journal of Molecular Sciences, 19(11), 3538 10.3390/ijms19113538 PMC6275013 30423996

[wrna1535-bib-0104] Lewis, B. P. , Burge, C. B. , & Bartel, D. P. (2005). Conserved seed pairing, often flanked by adenosines, indicates that thousands of human genes are microRNA targets. Cell, 120, 15–20. 10.1016/J.CELL.2004.12.035 15652477

[wrna1535-bib-0105] Lewis, B. P. , Shih, I. H. , Jones‐Rhoades, M. W. , Bartel, D. P. , & Burge, C. B. (2003). Prediction of mammalian microRNA targets. Cell, 115(7), 787–798. 14697198 10.1016/s0092-8674(03)01018-3

[wrna1535-bib-0106] Li, K. , Wong, D. K. , Hong, K. Y. , & Raffai, R. L. (2018). Cushioned‐density gradient ultracentrifugation (C‐DGUC): A refined and high performance method for the isolation, characterization, and use of exosomes. Methods in Molecular Biology, 1740, 69–83. 10.1007/978-1-4939-7652-2_7 29388137 PMC6476194

[wrna1535-bib-0107] Li, S. , Liu, L. , Zhuang, X. , Yu, Y. , Liu, X. , Cui, X. , … Chen, X. (2013). MicroRNAs inhibit the translation of target mRNAs on the endoplasmic reticulum in Arabidopsis. Cell, 153(3), 562–574. 10.1016/j.cell.2013.04.005 23622241 PMC3694718

[wrna1535-bib-0108] Li, S. , Xu, Z. , & Sheng, J. (2018). tRNA‐derived small RNA: A novel regulatory small non‐coding RNA. Genes, 9, 246 10.3390/GENES9050246 PMC5977186 29748504

[wrna1535-bib-0109] Libri, V. , Helwak, A. , Miesen, P. , Santhakumar, D. , Borger, J. G. , Kudla, G. , … Buck, A. H. (2012). Murine cytomegalovirus encodes a miR‐27 inhibitor disguised as a target. Proceedings of the National Academy of Sciences of the United States of America, 109, 279–284. 10.1073/pnas.1114204 22184245 PMC3252920

[wrna1535-bib-0110] Linde, M. E. , Colquhoun, D. R. , Ubaida Mohien, C. , Kole, T. , Aquino, V. , Cotter, R. , … Graham, D. R. (2013). The conserved set of host proteins incorporated into HIV‐1 Virions suggests a common egress pathway in multiple cell types. Journal of Proteome Research, 12, 2045–2054. 10.1021/pr300918r 23432411 PMC4065613

[wrna1535-bib-0111] Liu, X. , Happel, C. , & Ziegelbauer, J. M. (2017). Kaposi's sarcoma‐associated herpesvirus microRNAs target GADD45B to protect infected cells from cell cycle arrest and apoptosis. Journal of Virology, 91, e02045‐16 10.1128/JVI.02045-16 27852859 PMC5244352

[wrna1535-bib-0112] Lötvall, J. , Hill, A. F. , Hochberg, F. , Buzás, E. I. , Di Vizio, D. , Gardiner, C. , … Théry, C. (2014). Minimal experimental requirements for definition of extracellular vesicles and their functions: A position statement from the International Society for Extracellular Vesicles. Journal of Extracellular Vesicles, 3, 26913 10.3402/JEV.V3.26913 25536934 PMC4275645

[wrna1535-bib-0113] Lu, Y. , Qin, Z. , Wang, J. , Zheng, X. , Lu, J. , Zhang, X. , … Ma, J. (2017). Epstein–Barr virus miR‐BART6‐3p inhibits the RIG‐I pathway. Journal of Innate Immunity, 9, 574–586. 10.1159/000479749 28877527

[wrna1535-bib-0700] Maemura, T. , Fukuyama, S., Sugita, Y., Lopes, T. J. S., Nakao, T., Noda, T., & Kawaoka, Y. (2018). Lung‐derived exosomal miR‐483‐3p regulates the innate immune response to influenza virus infection. The Journal of Infectious Diseases, 217, 1372–1382. 10.1093/infdis/jiy035 29373693

[wrna1535-bib-0114] Mahajan, V. S. , Drake, A. , & Chen, J. (2009). Virus‐specific host miRNAs: Antiviral defenses or promoters of persistent infection? Trends in Immunology, 30, 1–7. 10.1016/j.it.2008.08.009 19059006 PMC2709239

[wrna1535-bib-0115] Mao, L. , Wu, J. , Shen, L. , Yang, J. , Chen, J. , & Xu, H. (2016). Enterovirus 71 transmission by exosomes establishes a productive infection in human neuroblastoma cells. Virus Genes, 52, 189–194. 10.1007/s11262-016-1292-3 26837894

[wrna1535-bib-0116] Marcinowski, L. , Tanguy, M. , Krmpotic, A. , Rädle, B. , Lisnić, V. J. , Tuddenham, L. , … Dölken, L. (2012). Degradation of cellular miR‐27 by a novel, highly abundant viral transcript is important for efficient virus replication in vivo. PLoS Pathogens, 8, e1002510 10.1371/journal.ppat.1002510 22346748 PMC3276556

[wrna1535-bib-0117] Masciopinto, F. , Giovani, C. , Campagnoli, S. , Galli‐Stampino, L. , Colombatto, P. , Brunetto, M. , … Abrignani, S. (2004). Association of hepatitis C virus envelope proteins with exosomes. European Journal of Immunology, 34, 2834–2842. 10.1002/eji.200424887 15368299

[wrna1535-bib-0118] Mateescu, B. , Kowal, E. J. K. , van Balkom, B. W. M. , Bartel, S. , Bhattacharyya, S. N. , Buzás, E. I. , … Nolte‐'t Hoen, E. N. M. (2017). Obstacles and opportunities in the functional analysis of extracellular vesicle RNA—an ISEV position paper. Journal of Extracellular Vesicles, 6, 1286095 10.1080/20013078.2017.1286095 28326170 PMC5345583

[wrna1535-bib-0119] McCaskill, J. , Praihirunkit, P. , Sharp, P. M. , & Buck, A. H. (2015). RNA‐mediated degradation of microRNAs: A widespread viral strategy? RNA Biology, 12, 579–585. 10.1080/15476286.2015.1034912 25849078 PMC4615357

[wrna1535-bib-0120] McKenzie, A. J. , Hoshino, D. , Hong, N. H. , Cha, D. J. , Franklin, J. L. , Coffey, R. J. , … Weaver, A. M. (2016). KRAS‐MEK signaling controls Ago2 sorting into exosomes. Cell Reports, 15(5), 978–987. 10.1016/j.celrep.2016.03.085 27117408 PMC4857875

[wrna1535-bib-0121] Meerloo, T. , Sheikh, M. A. , Bloem, A. C. , de Ronde, A. , Schutten, M. , van Els, C. A. , … Schuurman, H. J. (1993). Host cell membrane proteins on human immunodeficiency virus type 1 after in vitro infection of H9 cells and blood mononuclear cells. An immuno‐electron microscopic study. Journal of General Virology, 74(Pt. 1), 129–135. 10.1099/0022-1317-74-1-129 8093711

[wrna1535-bib-0122] Mendell, J. T. , & Olson, E. N. (2012). MicroRNAs in stress signaling and human disease. Cell, 148(6), 1172–1187. 10.1016/j.cell.2012.02.005 22424228 PMC3308137

[wrna1535-bib-0123] Mercer, J. , Schelhaas, M. , & Helenius, A. (2010). Virus entry by endocytosis. Annual Review of Biochemistry, 79, 803–833. 10.1146/annurev-biochem-060208-104626 20196649

[wrna1535-bib-0124] Mitchell, P. S. , Parkin, R. K. , Kroh, E. M. , Fritz, B. R. , Wyman, S. K. , Pogosova‐Agadjanyan, E. L. , … Tewari, M. (2008). Circulating microRNAs as stable blood‐based markers for cancer detection. Proceedings of the National Academy of Sciences, 105, 10513–10518. 10.1073/pnas.0804549105 PMC2492472 18663219

[wrna1535-bib-0125] Montecalvo, A. , Larregina, A. T. , Shufesky, W. J. , Stolz, D. B. , Sullivan, M. L. G. , Karlsson, J. M. , … Morelli, A. E. (2012). Mechanism of transfer of functional microRNAs between mouse dendritic cells via exosomes. Blood, 119, 756–766. 10.1182/blood-2011-02-338004 22031862 PMC3265200

[wrna1535-bib-0126] Morelli, A. E. , Larregina, A. T. , Shufesky, W. J. , Sullivan, M. L. G. , Stolz, D. B. , Papworth, G. D. , … Thomson, A. W. (2004). Endocytosis, intracellular sorting, and processing of exosomes by dendritic cells. Blood, 104, 3257–3266. 10.1182/blood-2004-03-0824 15284116

[wrna1535-bib-0127] Mori, Y. , Koike, M. , Moriishi, E. , Kawabata, A. , Tang, H. , Oyaizu, H. , … Yamanishi, K. (2008). Human herpesvirus‐6 induces MVB formation, and virus egress occurs by an exosomal release pathway. Traffic, 9, 1728–1742. 10.1111/j.1600-0854.2008.00796.x 18637904 PMC2613231

[wrna1535-bib-0128] Mukherjee, S. , Akbar, I. , Kumari, B. , Vrati, S. , Basu, A. , & Banerjee, A. (2018). Japanese encephalitis virus‐induced let‐7a/b interacted with the NOTCH‐TLR7 pathway in microglia and facilitated neuronal death via caspase activation. Journal of Neurochemistry. Epub ahead of print. 10.1111/jnc.14645 30556910

[wrna1535-bib-0129] Mulcahy, L. A. , Pink, R. C. , & Carter, D. R. F. (2014). Routes and mechanisms of extracellular vesicle uptake. Journal of Extracellular Vesicles, 3, 24641 10.3402/jev.v3.24641 PMC4122821 25143819

[wrna1535-bib-0130] Munshi, A. , Mohan, V. , & Ahuja, Y. (2016). Non‐coding RNAs: A dynamic and complex network of gene regulation. Journal of Pharmacogenomics & Pharmacoproteomics, 7, 2153–0645. 10.4172/2153-0645.1000156

[wrna1535-bib-0131] Nabet, B. Y. , Qiu, Y. , Shabason, J. E. , Wu, T. J. , Yoon, T. , Kim, B. C. , … Minn, A. J. (2017). Exosome RNA unshielding couples stromal activation to pattern recognition receptor signaling in cancer. Cell, 170, 352–366. 10.1016/J.CELL.2017.06.031 28709002 PMC6611169

[wrna1535-bib-0132] Nakase, I. , Kobayashi, N. B. , Takatani‐Nakase, T. , & Yoshida, T. (2015). Active macropinocytosis induction by stimulation of epidermal growth factor receptor and oncogenic Ras expression potentiates cellular uptake efficacy of exosomes. Scientific Reports, 5, 10300 10.1038/srep10300 26036864 PMC4453128

[wrna1535-bib-0133] Nanbo, A. , Kawanishi, E. , Yoshida, R. , & Yoshiyama, H. (2013). Exosomes derived from Epstein–Barr virus‐infected cells are internalized via caveola‐dependent endocytosis and promote phenotypic modulation in target cells. Journal of Virology, 87, 10334–10347. 10.1128/JVI.01310-13 23864627 PMC3753980

[wrna1535-bib-0134] Narayanan, A. , Iordanskiy, S. , Das, R. , Van Duyne, R. , Santos, S. , Jaworski, E. , … Kashanchi, F. (2013). Exosomes derived from HIV‐1‐infected cells contain trans‐activation response element RNA. Journal of Biological Chemistry, 288, 20014–20033. 10.1074/jbc.M112.438895 23661700 PMC3707700

[wrna1535-bib-0135] Narayanan, A. , Kehn‐Hall, K. , Bailey, C. , & Kashanchi, F. (2011). Analysis of the roles of HIV‐derived microRNAs. Expert Opinion on Biological Therapy, 11, 17–29. 10.1517/14712598.2011.540564 21133815

[wrna1535-bib-0136] Nolte‐'t Hoen, E. N. M. , Buermans, H. P. J. , Waasdorp, M. , Stoorvogel, W. , Wauben, M. H. M. , & 't Hoen, P. A. C. (2012). Deep sequencing of RNA from immune cell‐derived vesicles uncovers the selective incorporation of small non‐coding RNA biotypes with potential regulatory functions. Nucleic Acids Research, 40, 9272–9285. 10.1093/nar/gks658 22821563 PMC3467056

[wrna1535-bib-0137] Novellino, L. , Rossi, R. L. , Bonino, F. , Cavallone, D. , Abrignani, S. , Pagani, M. , & Brunetto, M. R. (2012). Circulating hepatitis B surface antigen particles carry hepatocellular microRNAs. PLoS One, 7, e31952 10.1371/journal.pone.0031952 22470417 PMC3314627

[wrna1535-bib-0138] Obbard, D. J. , Gordon, K. H. J. , Buck, A. H. , & Jiggins, F. M. (2009). The evolution of RNAi as a defence against viruses and transposable elements. Philosophical Transactions of the Royal Society of London Series B: Biological Sciences, 364, 99–115. 10.1098/rstb.2008.0168 18926973 PMC2592633

[wrna1535-bib-0139] Orentas, R. J. , & Hildreth, J. E. (1993). Association of host cell surface adhesion receptors and other membrane proteins with HIV and SIV. AIDS Research and Human Retroviruses, 9(11), 1157–1165. 10.1089/aid.1993.9.1157 8312057

[wrna1535-bib-0140] Ouellet, D. L. , Plante, I. , Landry, P. , Barat, C. , Janelle, M.‐E. , Flamand, L. , … Provost, P. (2008). Identification of functional microRNAs released through asymmetrical processing of HIV‐1 TAR element. Nucleic Acids Research, 36, 2353–2365. 10.1093/nar/gkn076 18299284 PMC2367715

[wrna1535-bib-0141] Ouyang, Y. , Bayer, A. , Chu, T. , Tyurin, V. A. , Kagan, V. E. , Morelli, A. E. , … Sadovsky, Y. (2016). Isolation of human trophoblastic extracellular vesicles and characterization of their cargo and antiviral activity. Placenta, 47, 86–95. 10.1016/J.PLACENTA.2016.09.008 27780544 PMC5123854

[wrna1535-bib-0142] Pare, J. M. , & Sullivan, C. S. (2014). Distinct antiviral responses in pluripotent versus differentiated cells. PLoS Pathogens, 10(2), e1003865 10.1371/journal.ppat.1003865 24516379 PMC3916405

[wrna1535-bib-0143] Parolini, I. , Federici, C. , Raggi, C. , Lugini, L. , Palleschi, S. , De Milito, A. , … Fais, S. (2009). Microenvironmental pH is a key factor for exosome traffic in tumor cells. Journal of Biological Chemistry, 284, 34211–34222. 10.1074/jbc.M109.041152 19801663 PMC2797191

[wrna1535-bib-0144] Pegtel, D. M. , Cosmopoulos, K. , Thorley‐Lawson, D. A. , van Eijndhoven, M. A. J. , Hopmans, E. S. , Lindenberg, J. L. , … Middeldorp, J. M. (2010). Functional delivery of viral miRNAs via exosomes. Proceedings of the National Academy of Sciences of the United States of America, 107, 6328–6333. 10.1073/pnas.0914843107 20304794 PMC2851954

[wrna1535-bib-0145] Pérez‐Boza, J. , Lion, M. , & Struman, I. (2018). Exploring the RNA landscape of endothelial exosomes. RNA, 24, 423–435. 10.1261/rna.064352.117 29282313 PMC5824360

[wrna1535-bib-0146] Pfeffer, S. , Zavolan, M. , Grässer, F. A. , Chien, M. , Russo, J. J. , Ju, J. , … Tuschl, T. (2004). Identification of virus‐encoded microRNAs. Science, 304(5671), 734–736. 10.1126/science.1096781 15118162

[wrna1535-bib-0147] Raab‐Traub, N. , & Dittmer, D. P. (2017). Viral effects on the content and function of extracellular vesicles. Nature Reviews. Microbiology, 15, 559–572. 10.1038/nrmicro.2017.60 28649136 PMC5555775

[wrna1535-bib-0148] Ramakrishnaiah, V. , Thumann, C. , Fofana, I. , Habersetzer, F. , Pan, Q. , de Ruiter, P. E. , … van der Laan, L. J. W. (2013). Exosome‐mediated transmission of hepatitis C virus between human hepatoma Huh7.5 cells. Proceedings of the National Academy of Sciences of the United States of America, 110, 13109–13113. 10.1073/pnas.1221899 23878230 PMC3740869

[wrna1535-bib-0149] Ridder, K. , Keller, S. , Dams, M. , Rupp, A.‐K. , Schlaudraff, J. , Del Turco, D. , … Momma, S. (2014). Extracellular vesicle‐mediated transfer of genetic information between the hematopoietic system and the brain in response to inflammation. PLoS Biology, 12, e1001874 10.1371/journal.pbio.1001874 24893313 PMC4043485

[wrna1535-bib-0150] Ridder, K. , Sevko, A. , Heide, J. , Dams, M. , Rupp, A.‐K. , Macas, J. , … Momma, S. (2015). Extracellular vesicle‐mediated transfer of functional RNA in the tumor microenvironment. OncoImmunology, 4, e1008371 10.1080/2162402X.2015.1008371 26155418 PMC4485784

[wrna1535-bib-0151] Rudt, S. , & Müller, R. H. (1993). In vitro phagocytosis assay of nano‐ and microparticles by chemiluminescence. III. Uptake of differently sized surface‐modified particles, and its correlation to particle properties and in vivo distribution. European Journal of Pharmaceutical Sciences, 1, 31–39. 10.1016/0928-0987(93)90015-3

[wrna1535-bib-0152] Ruggero, K. , Guffanti, A. , Corradin, A. , Sharma, V. K. , De Bellis, G. , Corti, G. , … D'Agostino, D. M. (2014). Small noncoding RNAs in cells transformed by human T‐cell leukemia virus type 1: A role for a tRNA fragment as a primer for reverse transcriptase. Journal of Virology, 88, 3612–3622. 10.1128/JVI.02823-13 24403582 PMC3993537

[wrna1535-bib-0153] Sampey, G. C. , Saifuddin, M. , Schwab, A. , Barclay, R. , Punya, S. , Chung, M.‐C. , … Kashanchi, F. (2016). Exosomes from HIV‐1‐infected cells stimulate production of pro‐inflammatory cytokines through trans‐activating response (TAR) RNA. Journal of Biological Chemistry, 291, 1251–1266. 10.1074/jbc.M115.662171 26553869 PMC4714213

[wrna1535-bib-0154] Santangelo, L. , Giurato, G. , Cicchini, C. , Montaldo, C. , Mancone, C. , Tarallo, R. , … Tripodi, M. (2016). The RNA‐binding protein SYNCRIP is a component of the hepatocyte exosomal machinery controlling microRNA sorting. Cell Reports, 17(3), 799–808. 10.1016/j.celrep.2016.09.031 27732855

[wrna1535-bib-0155] Sarnow, P. , & Sagan, S. M. (2016). Unraveling the mysterious interactions between hepatitis C virus RNA and liver‐specific microRNA‐122. Annual Review of Virology, 3, 309–332. 10.1146/annurev-virology-110615-042409 27578438

[wrna1535-bib-0156] Scheller, N. , Herold, S. , Kellner, R. , Bertrams, W. , Jung, A. L. , Janga, H. , … Schmeck, B. (2018). Proviral microRNAs detected in extracellular vesicles from bronchoalveolar lavage fluid of patients with influenza virus‐induced acute respiratory distress syndrome. Journal of Infectious Diseases, 219, 540–543. 10.1093/infdis/jiy554 30239899

[wrna1535-bib-0157] Schmieder, R. , Lim, Y. W. , & Edwards, R. (2012). Identification and removal of ribosomal RNA sequences from metatranscriptomes. Bioinformatics, 28, 433–435. 10.1093/bioinformatics/btr669 22155869 PMC3268242

[wrna1535-bib-0158] Schopman, N. C. T. , Willemsen, M. , Liu, Y. P. , Bradley, T. , van Kampen, A. , Baas, F. , … Haasnoot, J. (2012). Deep sequencing of virus‐infected cells reveals HIV‐encoded small RNAs. Nucleic Acids Research, 40, 414–427. 10.1093/nar/gkr719 21911362 PMC3245934

[wrna1535-bib-0159] Schorey, J. S. , Cheng, Y. , Singh, P. P. , & Smith, V. L. (2015). Exosomes and other extracellular vesicles in host–pathogen interactions. EMBO Reports, 16, 24–43. 10.15252/embr.201439363 25488940 PMC4304727

[wrna1535-bib-0160] Selitsky, S. R. , Baran‐Gale, J. , Honda, M. , Yamane, D. , Masaki, T. , Fannin, E. E. , … Sethupathy, P. (2015). Small tRNA‐derived RNAs are increased and more abundant than microRNAs in chronic hepatitis B and C. Scientific Reports, 5, 7675 10.1038/srep07675 25567797 PMC4286764

[wrna1535-bib-0161] Seo, G. J. , Kincaid, R. P. , Phanaksri, T. , Burke, J. M. , Pare, J. M. , Cox, J. E. , … Sullivan, C. S. (2013). Reciprocal inhibition between intracellular antiviral signaling and the RNAi machinery in mammalian cells. Cell Host & Microbe, 14, 435–445. 10.1016/J.CHOM.2013.09.002 24075860 PMC3837626

[wrna1535-bib-0162] Shelke, G. V. , Lässer, C. , Gho, Y. S. , & Lötvall, J. (2014). Importance of exosome depletion protocols to eliminate functional and RNA‐containing extracellular vesicles from fetal bovine serum. Journal of Extracellular Vesicles, 3, 24783 10.3402/jev.v3.24783 PMC4185091 25317276

[wrna1535-bib-0163] Shimakami, T. , Yamane, D. , Jangra, R. K. , Kempf, B. J. , Spaniel, C. , Barton, D. J. , & Lemon, S. M. (2012). Stabilization of hepatitis C virus RNA by an Ago2‐miR‐122 complex. Proceedings of the National Academy of Sciences of the United States of America, 109, 941–946. 10.1073/pnas.1112263109 22215596 PMC3271899

[wrna1535-bib-0164] Skog, J. , Würdinger, T. , van Rijn, S. , Meijer, D. H. , Gainche, L. , Curry, W. T. , … Breakefield, X. O. (2008). Glioblastoma microvesicles transport RNA and proteins that promote tumour growth and provide diagnostic biomarkers. Nature Cell Biology, 10, 1470–1476. 10.1038/ncb1800 19011622 PMC3423894

[wrna1535-bib-0165] Sohel, M. H. (2016). Extracellular/circulating microRNAs: Release mechanisms, functions and challenges. Achievements in the Life Sciences, 10, 175–186. 10.1016/J.ALS.2016.11.007

[wrna1535-bib-0166] Stalder, L. , Heusermann, W. , Sokol, L. , Trojer, D. , Wirz, J. , Hean, J. , … Meisner‐Kober, N. C. (2013). The rough endoplasmatic reticulum is a central nucleation site of siRNA‐mediated RNA silencing. EMBO Journal, 32(8), 1115–1127. 10.1038/emboj.2013.52 23511973 PMC3630355

[wrna1535-bib-0167] Stroun, M. , Anker, P. , Beljanski, M. , Henri, J. , Lederrey, C. , Ojha, M. , & Maurice, P. A. (1978). Presence of RNA in the nucleoprotein complex spontaneously released by human lymphocytes and frog auricles in culture. Cancer Research, 38, 3546–3554. 688240

[wrna1535-bib-0168] Sullivan, C. S. , Grundhoff, A. T. , Tevethia, S. , Pipas, J. M. , & Ganem, D. (2005). SV40‐encoded microRNAs regulate viral gene expression and reduce susceptibility to cytotoxic T cells. Nature, 435, 682–686. 10.1038/nature03576 15931223

[wrna1535-bib-0169] Svensson, K. J. , Christianson, H. C. , Wittrup, A. , Bourseau‐Guilmain, E. , Lindqvist, E. , Svensson, L. M. , … Belting, M. (2013). Exosome uptake depends on ERK1/2‐heat shock protein 27 signaling and lipid raft‐mediated endocytosis negatively regulated by caveolin‐1. Journal of Biological Chemistry, 288, 17713–17724. 10.1074/jbc.M112.445403 23653359 PMC3682571

[wrna1535-bib-0170] tenOever, B. R. (2017). Questioning antiviral RNAi in mammals. Nature Microbiology, 2, 17052 10.1038/nmicrobiol.2017.52 28440277

[wrna1535-bib-0171] Théry, C. , Amigorena, S. , Raposo, G. , & Clayton, A. (2006). Isolation and characterization of exosomes from cell culture supernatants and biological fluids. Current Protocols in Cell Biology, 30, 3.22.21–3.22.29. 10.1002/0471143030.cb0322s30 18228490

[wrna1535-bib-0172] Théry, C. , Witwer, K. W. , Aikawa, E. , Alcaraz, M. J. , Anderson, J. D. , Andriantsitohaina, R. , … Zuba‐Surma, E. K. (2018). Minimal information for studies of extracellular vesicles 2018 (MISEV2018): A position statement of the International Society for Extracellular Vesicles and update of the MISEV2014 guidelines. Journal of Extracellular Vesicles, 7(1), 1535750 10.1080/20013078.2018.1535750 30637094 PMC6322352

[wrna1535-bib-0173] Thompson, A. I. , Conroy, K. P. , & Henderson, N. C. (2015). Hepatic stellate cells: Central modulators of hepatic carcinogenesis. BMC Gastroenterology, 15, 63 10.1186/s12876-015-0291-5 26013123 PMC4445994

[wrna1535-bib-0174] Tian, T. , Zhu, Y.‐L. , Zhou, Y.‐Y. , Liang, G.‐F. , Wang, Y.‐Y. , Hu, F.‐H. , & Xiao, Z.‐D. (2014). Exosome uptake through clathrin‐mediated endocytosis and macropinocytosis and mediating miR‐21 delivery. Journal of Biological Chemistry, 289, 22258–22267. 10.1074/jbc.M114.588046 24951588 PMC4139237

[wrna1535-bib-0175] Tosar, J. P. , Cayota, A. , Eitan, E. , Halushka, M. K. , Witwer, K. W. , & Schiffelers, R. (2017). Ribonucleic artefacts: Are some extracellular RNA discoveries driven by cell culture medium components? Journal of Extracellular Vesicles, 6, 1272832. 10.1080/20013078.2016.1272832 PMC5328325 28326168

[wrna1535-bib-0176] Tosar, J. P. , Gámbaro, F. , Darré, L. , Pantano, S. , Westhof, E. , & Cayota, A. (2018). Dimerization confers increased stability to nucleases in 5′ halves from glycine and glutamic acid tRNAs. Nucleic Acids Research, 46, 9081–9093. 10.1093/nar/gky495 29893896 PMC6158491

[wrna1535-bib-0177] Tosar, J. P. , Gambaro, F. , Sanguinetti, J. , Bonilla, B. , Witwer, K. W. , & Cayota, A. (2015). Assessment of small RNA sorting into different extracellular fractions revealed by high‐throughput sequencing of breast cell lines. Nucleic Acids Research, 43, 5601–5616. 10.1093/nar/gkv432 25940616 PMC4477662

[wrna1535-bib-0178] Trobaugh, D. W. , Gardner, C. L. , Sun, C. , Haddow, A. D. , Wang, E. , Chapnik, E. , … Klimstra, W. B. (2014). RNA viruses can hijack vertebrate microRNAs to suppress innate immunity. Nature, 506, 245–248. 10.1038/nature12869 24352241 PMC4349380

[wrna1535-bib-0179] Trobaugh, D. W. , & Klimstra, W. B. (2017). MicroRNA regulation of RNA virus replication and pathogenesis. Trends in Molecular Medicine, 23, 80–93. 10.1016/J.MOLMED.2016.11.003 27989642 PMC5836316

[wrna1535-bib-0180] Tycowski, K. T. , Guo, Y. E. , Lee, N. , Moss, W. N. , Vallery, T. K. , Xie, M. , & Steitz, J. A. (2015). Viral noncoding RNAs: More surprises. Genes & Development, 29, 567–584. 10.1101/gad.259077.115 25792595 PMC4378190

[wrna1535-bib-0181] Valadi, H. , Ekström, K. , Bossios, A. , Sjöstrand, M. , Lee, J. J. , & Lötvall, J. O. (2007). Exosome‐mediated transfer of mRNAs and microRNAs is a novel mechanism of genetic exchange between cells. Nature Cell Biology, 9, 654–659. 10.1038/ncb1596 17486113

[wrna1535-bib-0182] van der Grein, S. G. , Defourny, K. A. Y. , Slot, E. F. J. , & Nolte‐'t Hoen, E. N. M. (2018). Intricate relationships between naked viruses and extracellular vesicles in the crosstalk between pathogen and host. Seminars in Immunopathology, 40, 491–504. 10.1007/s00281-018-0678-9 29789863 PMC6208671

[wrna1535-bib-0183] van der Grein, S. G. , & Nolte‐'t Hoen, E. N. (2014). "Small talk" in the innate immune system via RNA‐containing extracellular vesicles. Frontiers in Immunology, 5, 542 10.3389/fimmu.2014.00542 25400635 PMC4212677

[wrna1535-bib-0184] van Dongen, H. M. , Masoumi, N. , Witwer, K. W. , & Pegtel, D. M. (2016). Extracellular vesicles exploit viral entry routes for cargo delivery. Microbiology and Molecular Biology Reviews, 80, 369–386. 10.1128/MMBR.00063-15 26935137 PMC4867369

[wrna1535-bib-0185] Villarroya‐Beltri, C. , Gutiérrez‐Vázquez, C. , Sánchez‐Cabo, F. , Pérez‐Hernández, D. , Vázquez, J. , Martin‐Cofreces, N. , … Sánchez‐Madrid, F. (2013). Sumoylated hnRNPA2B1 controls the sorting of miRNAs into exosomes through binding to specific motifs. Nature Communications, 4, 2980 10.1038/ncomms3980 PMC3905700 24356509

[wrna1535-bib-0186] Wang, Q. , Lee, I. , Ren, J. , Ajay, S. S. , Lee, Y. S. , & Bao, X. (2013). Identification and functional characterization of tRNA‐derived RNA fragments (tRFs) in respiratory syncytial virus infection. Molecular Therapy, 21, 368–379. 10.1038/mt.2012.237 23183536 PMC3594034

[wrna1535-bib-0187] Wang, W. , Li, J. , Zhang, X. , Wen, Y. , Wang, X.‐Y. , & Yuan, Z. (2016). A pilot study of microRNAs expression profile in serum and HBsAg particles. Medicine, 95, e2511 10.1097/MD.0000000000002511 26765470 PMC4718296

[wrna1535-bib-0188] Wang, Y. , Li, H. , Sun, Q. , & Yao, Y. (2016). Characterization of small RNAs derived from tRNAs, rRNAs and snoRNAs and their response to heat stress in wheat seedlings. PLoS One, 11, e0150933 10.1371/journal.pone.0150933 26963812 PMC4786338

[wrna1535-bib-0189] Watanabe, T. , Sorensen, E. M. , Naito, A. , Schott, M. , Kim, S. , & Ahlquist, P. (2007). Involvement of host cellular multivesicular body functions in hepatitis B virus budding. Proceedings of the National Academy of Sciences of the United States of America, 104(24), 10205–10210. 10.1073/pnas.0704000104 17551004 PMC1891263

[wrna1535-bib-0190] Wei, Z. , Batagov, A. O. , Carter, D. R. F. , & Krichevsky, A. M. (2016). Fetal bovine serum RNA interferes with the cell culture derived extracellular RNA. Scientific Reports, 6, 31175 10.1038/srep31175 27503761 PMC4977539

[wrna1535-bib-0191] Wei, Z. , Batagov, A. O. , Schinelli, S. , Wang, J. , Wang, Y. , El Fatimy, R. , … Krichevsky, A. M. (2017). Coding and noncoding landscape of extracellular RNA released by human glioma stem cells. Nature Communications, 8, 1145 10.1038/s41467-017-01196-x PMC5658400 29074968

[wrna1535-bib-0192] Witteveldt, J. , Ivens, A. , & Macias, S. (2018). Inhibition of microprocessor function during the activation of the type I interferon response. Cell Reports, 23, 3275–3285. 10.1016/j.celrep.2018.05.049 29898398 PMC6019736

[wrna1535-bib-0193] Witwer, K. W. , Sisk, J. M. , Gama, L. , & Clements, J. E. (2010). MicroRNA regulation of IFN‐β protein expression: Rapid and sensitive modulation of the innate immune response. Journal of Immunology, 184, 2369–2376. 10.4049/JIMMUNOL.0902712 PMC3076721 20130213

[wrna1535-bib-0194] Wu, M. , Ouyang, Y. , Wang, Z. , Zhang, R. , Huang, P.‐H. , Chen, C. , … Huang, T. J. (2017). Isolation of exosomes from whole blood by integrating acoustics and microfluidics. Proceedings of the National Academy of Sciences of the United States of America, 114, 10584–10589. 10.1073/pnas.1709210 28923936 PMC5635903

[wrna1535-bib-0195] Wu, S. , He, L. , Li, Y. , Wang, T. , Feng, L. , Jiang, L. , … Huang, X. (2013). miR‐146a facilitates replication of dengue virus by dampening interferon induction by targeting TRAF6. Journal of Infection, 67, 329–341. 10.1016/J.JINF.2013.05.003 23685241

[wrna1535-bib-0196] Xie, M. , & Steitz, J. A. (2014). Versatile microRNA biogenesis in animals and their viruses. RNA Biology, 11, 673–681. 10.4161/RNA.28985 24823351 PMC4156499

[wrna1535-bib-0197] Yáñez‐Mó, M. , R‐M Siljander, P. , Andreu, Z. , Bedina Zavec, A. , Borràs, F. E. , Buzas, E. I. , … De Wever, O. (2015). Biological properties of extracellular vesicles and their physiological functions. Journal of Extracellular Vesicles, 4, 27066 10.3402/jev.v4.27066 25979354 PMC4433489

[wrna1535-bib-0198] Yeung, M. L. , Bennasser, Y. , Watashi, K. , Le, S.‐Y. , Houzet, L. , & Jeang, K.‐T. (2009). Pyrosequencing of small non‐coding RNAs in HIV‐1 infected cells: Evidence for the processing of a viral‐cellular double‐stranded RNA hybrid. Nucleic Acids Research, 37, 6575–6586. 10.1093/nar/gkp707 19729508 PMC2770672

[wrna1535-bib-0199] Yin, C. , Evason, K. J. , Asahina, K. , & Stainier, D. Y. (2013). Hepatic stellate cells in liver development, regeneration, and cancer. Journal of Clinical Investigation, 123(5), 1902–1910. 10.1172/JCI66369 23635788 PMC3635734

[wrna1535-bib-0200] Yogev, O. , Henderson, S. , Hayes, M. J. , Marelli, S. S. , Ofir‐Birin, Y. , Regev‐Rudzki, N. , … Enver, T. (2017). Herpesviruses shape tumour microenvironment through exosomal transfer of viral microRNAs. PLoS Pathogens, 13, e1006524 10.1371/journal.ppat.1006524 28837697 PMC5570218

[wrna1535-bib-0201] Yoshida, R. , Takaesu, G. , Yoshida, H. , Okamoto, F. , Yoshioka, T. , Choi, Y. , … Kobayashi, T. (2008). TRAF6 and MEKK1 play a pivotal role in the RIG‐I‐like helicase antiviral pathway. Journal of Biological Chemistry, 283, 36211–36220. 10.1074/jbc.M806576200 18984593 PMC2662295

[wrna1535-bib-0202] Zhang, Y. , Zhang, Y. , Shi, J. , Zhang, H. , Cao, Z. , Gao, X. , … Duan, E. (2014). Identification and characterization of an ancient class of small RNAs enriched in serum associating with active infection. Journal of Molecular Cell Biology, 6, 172–174. 10.1093/jmcb/mjt052 24380870

[wrna1535-bib-0203] Zhou, D. , Xue, J. , He, S. , Du, X. , Zhou, J. , Li, C. , … Cheng, Z. (2018). Reticuloendotheliosis virus and avian leukosis virus subgroup J synergistically increase the accumulation of exosomal miRNAs. Retrovirology, 15, 45 10.1186/s12977-018-0427-0 29970099 PMC6029113

[wrna1535-bib-0204] Zhou, Y. , Wang, X. , Sun, L. , Zhou, L. , Ma, T.‐C. , Song, L. , … Ho, W.‐Z. (2016). Toll‐like receptor 3‐activated macrophages confer anti‐HCV activity to hepatocytes through exosomes. FASEB Journal, 30, 4132–4140. 10.1096/fj.201600696R 27605546 PMC5102108

[wrna1535-bib-0205] Zomer, A. , Maynard, C. , Verweij, F. J. , Kamermans, A. , Schäfer, R. , Beerling, E. , … van Rheenen, J. (2015). Vivo imaging reveals extracellular vesicle‐mediated phenocopying of metastatic behavior. Cell, 161, 1046–1057. 10.1016/J.CELL.2015.04.042 26000481 PMC4448148

[wrna1535-bib-0206] Zomer, A. , Steenbeek, S. C. , Maynard, C. , & van Rheenen, J. (2016). Studying extracellular vesicle transfer by a Cre‐loxP method. Nature Protocols, 11, 87–101. 10.1038/nprot.2015.138 26658469

